# IRF1-mediated upregulation of PARP12 promotes cartilage degradation by inhibiting PINK1/Parkin dependent mitophagy through ISG15 attenuating ubiquitylation and SUMOylation of MFN1/2

**DOI:** 10.1038/s41413-024-00363-3

**Published:** 2024-10-28

**Authors:** Zengfa Deng, Dianbo Long, Changzhao Li, Hailong Liu, Wei Li, Yanlin Zhong, Xiaolin Mo, Ruiyun Li, Zibo Yang, Yan Kang, Guping Mao

**Affiliations:** 1 Department of Sports Medicine, the First Affiliated Hospital, Sun Yat-sen University, Guangzhou, Guangdong 510080 China; 2 Guangdong Provincial Key Laboratory of Orthopedics and Traumatology, the First Affiliated Hospital, Sun Yat-sen University, Guangzhou, Guangdong 510080 China; 3 Department of Joint Surgery and Sports Medicine, Center for Orthopedic Surgery, The Third Affiliated Hospital of Southern Medical University, Guangzhou, Guangdong China; 4 Department of Joint Surgery, the First Affiliated Hospital, Sun Yat-sen University, Guangzhou, Guangdong, 510080, China; 5 Department of Anesthesiology, Guangdong Provincial People’s Hospital (Guangdong Academy of Medical Sciences), Southern Medical University, Guangzhou, China

**Keywords:** Mitochondria, Homeostasis

## Abstract

Osteoarthritis (OA) is an age-related cartilage-degenerating joint disease. Mitochondrial dysfunction has been reported to promote the development of OA. Poly (ADP-ribose) polymerase family member 12 (PARP12) is a key regulator of mitochondrial function, protein translation, and inflammation. However, the role of PARP12 in OA-based cartilage degradation and the underlying mechanisms are relatively unknown. Here, we first demonstrated that PARP12 inhibits mitophagy and promotes OA progression in human OA cartilage and a monosodium iodoacetate-induced rat OA model. Using mass spectrometry and co-immunoprecipitation assay, PARP12 was shown to interact with ISG15, upregulate mitofusin 1 and 2 (MFN1/2) ISGylation, which downregulated MFN1/2 ubiquitination and SUMOylation, thereby inhibiting PINK1/Parkin-dependent chondrocyte mitophagy and promoting cartilage degradation. Moreover, inflammatory cytokine-induced interferon regulatory factor 1 (IRF1) activation was required for the upregulation of PARP12 expression, and it directly bound to the PARP12 promoter to activate transcription. XAV-939 inhibited PARP12 expression and suppressed OA pathogenesis in vitro and in vivo. Clinically, PARP12 can be used to predict the severity of OA; thus, it represents a new target for the study of mitophagy and OA progression. In brief, the IRF1-mediated upregulation of PARP12 promoted cartilage degradation by inhibiting PINK1/Parkin-dependent mitophagy via ISG15-based attenuation of MFN1/2 ubiquitylation and SUMOylation. Our data provide new insights into the molecular mechanisms underlying PARP12-based regulation of mitophagy and can facilitate the development of therapeutic strategies for the treatment of OA.

## Introduction

Osteoarthritis (OA) is a common chronic joint disease with clinical manifestations that include pain, stiffness, dysfunction, and deformities, which seriously limit the quality of work and life of patients.^1,2^ The development of OA involves multiple pathological changes in joints, including cartilage degeneration, osteophyte formation, synovial inflammation, and meniscus degeneration. However, cartilage degeneration is the landmark change,^3^ and it is driven by the increased catabolism and decreased anabolism of the extracellular matrix (ECM) by chondrocytes.^4,5^ The mechanism underlying the imbalance between catabolism and anabolism of the ECM by chondrocytes has been associated with many factors, including decreased autophagy, mitochondrial dysfunction, oxidative stress, and aging.^6,7^ Autophagy is a basic cellular process that maintains cell metabolism by degrading dysfunctional organelles and macromolecules, and it is important for the promotion of cell homeostasis, differentiation, development, and survival.^8^ Many patients with OA have been reported to exhibit mitochondrial dysfunction.^9,10^ Mitophagy, a selective autophagy, is spontaneously induced when mitochondria are damaged or dysfunctional; it protects cells by removing these cytotoxic stimuli.^11–13^ Recent studies found that drug-induced increases in the level of mitophagy in chondrocytes inhibit the development of OA.^14,15^ However, the molecular mechanisms underlying mitophagy in OA require further exploration.

The poly-ADP-ribosyl polymerase (PARP) family includes enzymes (17 PARP members in humans) that regulate various cellular responses, such as DNA repair and replication, metabolic regulation, and inflammation, through the modification of target proteins with an ADP-ribose using nicotinamide adenine dinucleotide (NAD^+^) as a substrate.^16–18^ Previous studies also indicated that PARP12 was an active enzyme that contributed to NAD^+^ consumption in the regulation of diverse cell processes, including immune responses and stress.^16,19^ A recent study found that LPAR5 promoted inflammatory process after stroke by enhancing microglia’s production of reactive oxygen species and nitric oxide synthase 2, and PARP14 promoted the level of autophagy of microglia by downregulating LPAR5 to improve functional recovery after stroke.^20^ Previous studies have shown that most members of PARPs are localized in the nucleus and their enzymatic activity mainly accounts for nuclear modifications.^21^ Unlike many other PARP members, PARP12 has been shown to be essentially distributed outside the nucleus and localized to distinct cytoplasmic structures in a protein domain-dependent manner. Interestingly, PARP12 has been reported to colocalize with p62,^22^ which might be associated with autophagy. However, the role of PARP12 in autophagy has not been reported. In addition, recent studies have enlarged our horizon, implying that PARP12 functions in regulating intracellular environmental homeostasis and disease development as well.^23,24^ However, the function of PARP12 in OA progression remains largely unknown.

Recently, post-translational modifications (PTMs) of proteins have been reported to play a significant role in maintaining protein homeostasis and in the development of diseases.^25^ Among these, ubiquitination and SUMOylation have garnered increasing attention.^26,27^ Ubiquitination involves the attachment of ubiquitin (a small molecular protein) to the target protein, which typically marks the protein for degradation or affects its function, localization, and interactions.^28^ SUMOylation is a process similar to ubiquitination, involving the attachment of small ubiquitin-related modifiers (SUMO) to proteins, which can regulate the stability, subcellular localization, function, and interactions of proteins with other proteins or DNA.^29^ Studies also indicated that ubiquitination and SUMOylation play multifaceted roles in the pathogenesis of OA.^30,31^ For instance, the dysregulation of ubiquitination and deubiquitylation processes may lead to an imbalance in articular cartilage, promoting the OA progression.^30^ Although the roles of ubiquitination and SUMOylation in OA are increasingly being recognized, the specific mechanisms of these modifications in the disease still require further investigation.

Interferon-stimulated gene 15 (ISG15) is a 15 kD protein that is involved in a ubiquitin-like post-translational process designated ISGylation through its conjugation to cellular proteins by ISG15-specific ligation enzymes.^32^ Recent studies indicated that ISG15 and ISGylation are associated with various diseases. For example, ISG15 and ISGylation is required for pancreatic cancer stem cell mitophagy and metabolic plasticity.^33^ ISGylation inhibits an LPS-induced inflammatory response via the TLR4/NF-κB signalling pathway in goat endometrial epithelial cells.^34^ However, the molecular functions and mechanisms of ISG15 and ISGylation in cartilage degradation remain unclear.

Mitofusin 1 and 2 (MFN1/2) are paralogous proteins that constitute members of the extensive superfamily of mitochondrial transmembrane GTPases, exhibiting partial functional overlap in the mechanism of mitochondrial fusion. MFN1/2 affects mitochondrial metabolism by regulating several mitochondrial functions such as membrane potential, fuel oxidation, and the OXPHOS system.^35,36^ In recent years, a study has indicated that overexpression of MFN2 played a pro-inflammatory role in DMM models by upregulating the expression of inflammation-related genes, including COX2 and MMP13, through the NF-κB pathway, and that knockdown of MFN2 had a protective effect against OA progression.^37^ Moreover, another study has also found that MFN2 overexpression exacerbated inflammation and chondrocyte apoptosis, leading to cartilage degeneration and OA progression.^38^ Additionally, there have been studies that reported MFN1/2 could inhibit mitophagy, thereby hindering the clearance of damaged mitochondria.^39,40^

In this study, we investigated the functions and molecular mechanisms of PAPR12 in OA in vitro and in vivo. Our study also revealed that PARP12 inhibits PINK1/Parkin-dependent mitophagy by upregulating the ISGylation of mitofusin 1 and 2 (MFN1/2) to promote cartilage degradation. In addition, we found that XAV-939 inhibits PARP12 expression and suppresses OA pathogenesis in vitro and in vivo, which may provide opportunities for the further development of OA treatments in future clinical trials. Moreover, we further found that PARP12 was upregulated by interferon regulatory factor 1 (IRF1)-associated inflammatory cytokines. Overall, these findings suggest that PARP12 may serve as a potential therapeutic target for OA treatment.

## Results

### PARP12 expression was significantly upregulated in damaged areas of knee OA cartilage

**Fig. 1:**
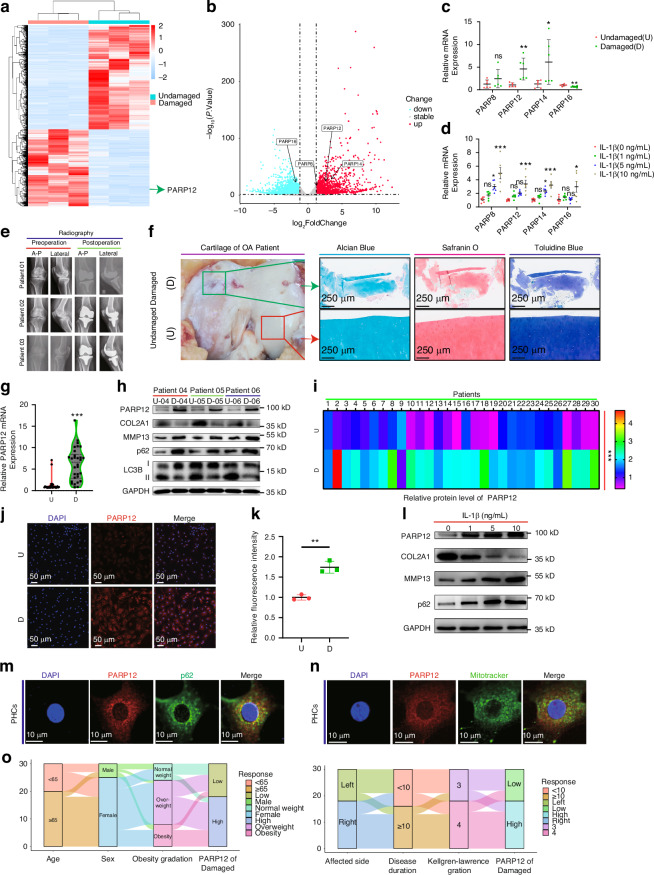
Upregulation of PARP12 in the damaged area of articular cartilage in patients with knee osteoarthritis (OA) and characterization of PARP12 in primary human chondrocytes (PHCs). **a** Heatmap of all differentially expressed genes between undamaged (U) and damaged (D) cartilage tissues of patients with OA. **b** Volcano plots of all differentially expressed genes, including the PARP8, PARP12, PARP14, and PARP16 members of the PARP superfamily, between U and D cartilage tissues. **c**, **d** Quantitative PCR analysis of PARP8, PARP12, PARP14, and PARP16 between U and D cartilage tissues and in PHCs treated with different concentrations of IL-1β (0, 1, 5, or 10 ng/mL) for 24 h. *n* = 6 per group. **e** Plain radiographs of patients with knee OA. **f** Representative image of intraoperative specimens and alcian blue, safranin O, and toluidine blue staining of U and D cartilage tissues. Scale bar: 250 µm. **g** Quantitative PCR analysis of PARP12 in U and D cartilage tissues. *n* = 30 per group. **h** Western blot analysis of PARP12, COL2A1, MMP13, p62, and LC3B between U and D cartilage tissues. **i** Heatmap of relative protein level of PARP12 in U and D cartilage tissues of different patients with OA. *n* = 30 per group. **j** Immunofluorescence of PARP12 in U and D cartilage tissues. Scale bars: 50 µm. **k** Immunofluorescence quantification of (**j**) using ImageJ. *n* = 3 per group. **L** Western blot analysis of PARP12, COL2A1, MMP13 and p62 in primary chondrocytes (PHCs) treated with different concentrations of IL-1β (0, 1, 5, or 10 ng/mL) for 24 h. **m** Colocalization of PARP12 and p62 in PHCs. Scale bars: 10 µm. **n** Colocalization of PARP12 and mitotracker in PHCs. Scale bars: 10 µm. **o** Relationship between age, sex, obesity gradation, affected side, disease duration, Kellgren–Lawrence gradation, and expression of PARP12 in the damaged area represented by the Sankey diagram. Scale bars: 10 µm. Data are presented as the mean ± SD. Paired t-test (**c**, **d**, **g**, **i**, **l**) was used for statistical analysis. *P < 0.05, **P < 0.01, and ***P < 0.001

Initially, we performed mRNA-seq analyses on chondrocytes of undamaged (U) and damaged (D) areas of three knee OA samples (GEO accession number GSE220487). Differentially expressed genes (2 041 upregulated and 2 769 downregulated) between the two chondrocyte populations (U vs. D chondrocytes) were identified (Fig. 1a). The expression of PARP8, PARP12, PARP14, and PARP16 from the PARP superfamily differed significantly between the U and D chondrocytes (Fig. 1b). We further verified the expression levels of these genes in U and D chondrocytes and primary human chondrocytes (PHCs) treated with different concentrations of interleukin-1β (IL-1β) using quantitative reverse transcription PCR (RT-qPCR). In our experiments, we found that PARP8 levels did not differ significantly between damaged (D) and undamaged (U) cartilage areas, while PARP12 and PARP14 levels were both significantly upregulated in the D areas compared to U areas (Fig. 1c). However, there was a high degree of variability in PARP14 expression, with some samples showing lower expression in the D areas than in the U areas of other samples. In contrast, PARP12 consistently showed higher expression in the D areas, with a correspondingly smaller *p*-value. The expression of PARP16 was significantly downregulated in the damaged areas but was upregulated in PHCs following treatment with IL-1β (10 ng/mL), indicating that PARP16 expression is relatively unstable in OA (Fig. 1c, d). We have further examined the protein levels of PARP16 in both D and U areas and found no statistically significant differences (Fig. S1A, B). Therefore, we did not conduct further research on PARP16. Overall, PARP12 consistently showed significantly higher expression in the D areas compared to the U areas, and its expression was notably upregulated in PHCs following treatment with IL-1β (10 ng/mL). Therefore, we chose to focus our research on PARP12.

Cartilage samples from 30 patients with knee OA were divided into U and D areas based on X-ray imaging and confirmed by staining of surgical specimens (Fig. 1e, f). We observed that the mRNA and protein levels of PARP12 were dramatically increased in D areas (Fig. 1g–k). In addition, the mRNA and protein levels of COL2A1 and MMP13 were downregulated and upregulated in D areas, respectively, while autophagy marker LC3B and p62 were downregulated and upregulated in D areas, respectively (Fig. 1h and Fig. S1C–F), as revealed by RT-qPCR, western blots and immunohistochemistry (IHC) staining, indicating deficient autophagy in the degenerative cartilage. We further confirmed the protein levels of PARP12, COL2A1, MMP13, and p62 in PHCs treated with different concentrations of IL-1β using western blots (Figs. 1l and S1G). Based on the above significant differences between treatments with IL-1β and control PHCs, we considered that 10 ng/mL of IL-1β was appropriate for the stimulation of PHCs. In addition, inflammatory stimulation upregulated PARP12 expression in chondrocytes but downregulated the autophagy ability. Finally, we also performed immunofluorescence in PHCs and found that PARP12 was mainly distributed in the cytoplasm and colocalized with p62 and mitotracker (Fig. 1m, n). Furthermore, the results of immunofluorescence showed that both the immunofluorescence co-localization levels of PARP12 with p62 and mitotracker were significantly upregulated in PHCs treated with IL-1β compared with the control group (Fig. S1H–K). These results showed that PARP12 was tightly correlated with mitophagy and OA progression.

To further explore the clinical significance of PARP12 expression in OA, we analysed the relationships between patient characteristics and protein levels of PARP12 in D areas using a Sankey diagram (Fig. 1o). Among 30 patients with knee OA, we found that 12 and 18 patients had low and high expression of PARP12 in D cartilage, respectively (Table 1). Chi-square test results indicated that high PARP12 expression levels were associated with disease duration (*P* = 0.024) and Kellgren-Lawrence gradation (*P* = 0.024) (Table 2). In addition, Spearman’s correlation analysis showed that high levels of PARP12 were correlated with age (*P* = 0.008), body mass index (BMI, *P* = 0.06), disease duration (*P* = 0.027), and Kellgren-Lawrence gradation (*P* < 0.001) (Table S1). These results indicated that PARP12 is correlated with clinical characteristics, including age, BMI, disease duration, and Kellgren-Lawrence gradation. Cumulatively, these results indicated that PARP12 was upregulated in D areas of knee OA cartilage and correlated with clinical characteristics, including age, BMI, disease duration, and Kellgren-Lawrence gradation; thus, it might contribute to OA progression.

**Table 1:** Baseline characteristics of patients with knee osteoarthritis (*n* = 30)

Characteristics	Number of cases (%)
Age/year	
< 65	10(33.3)
≥ 65	20(66.7)
Gender	
Male	5(16.7)
Female	25(83.3)
Obesity gradation^a^	
Underweight	0(0.0)
Normal weight	6(20.0)
Overweight	16(53.3)
Obesity	8(26.7)
Affected side	
Left	12(40)
Right	18(60)
Disease duration/year	
< 10	14(46.7)
≥ 10	16(53.3)
Kellgren–Lawrence gradation	
III	12(40)
IV	18(60)
Expression of PARP12^b^	
Low expression	12(40)
High expression	18(60)

^a^Underweight: BMI < 18.5; Normal weight: 18.5 ≤ BMI < 24; Overweight: 24 ≤ BMI < 28; Obesity: BMI ≥ 28

^b^The maximal difference (thirteenth minus twelfth is 0.065 4 in ascending order) near median was used to classify between the low expression or high expression of PARP12 instead of the median (sixteenth minus fifteenth is 0.000 7 in ascending order)

**Table 2:** Correlation between PARP12 expression and the baseline characteristics of patients with knee osteoarthritis (*n* = 30)

Characteristics	PARP12 expression		P*
	Low	High	
Age/year			
<65	5	5	0.461
≥ 65	7	13	
Gender			
Male	3	2	0.364
Female	9	16	
Obesity gradation			
Underweight	0	0	0.290
Normal weight	4	2	
Overweight	6	10	
Obesity	2	6	
Affected side			
Left	5	7	1.000
Right	7	11	
Disease duration/year			
<10	9	5	0.024
≥ 10	3	13	
Kellgren–Lawrence gradation			
III	8	4	0.024
IV	4	14	

^*^P values were analyzed using chi-square test with *P* <0.05 as significant

### PARP12 was upregulated in rat articular cartilage and chondrocytes with OA

**Fig. 2:**
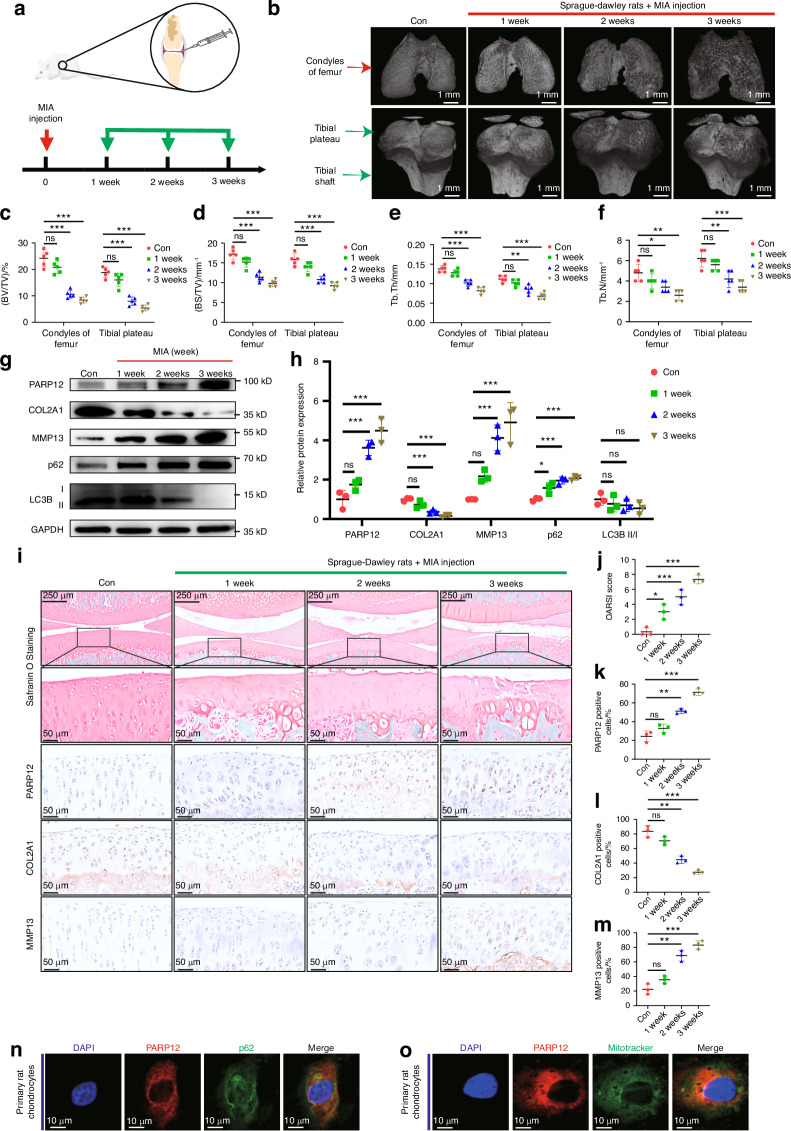
PARP12 is upregulated in rat OA chondrocytes and rat articular cartilage. **a** Experimental diagram of the OA rat model induced by the injection of 40 μL saline with or without 1 mg MIA into the intraarticular cavity of 8-week-old Sprague–Dawley rats for 1, 2, or 3 weeks. *n* = 6 per group. **b** 3D reconstruction images of micro-CT scanning. *n* = 5 per group. **c**–**f** Analysis of bone volume (BV)/trabecular volume (TV) ratio, bone surface (BS)/TV ratio, trabecular thickness, and trabecular numbers. *n* = 5 per group. **g** Western blot analysis of PARP12, COL2A1, MMP13, p62, and LC3B in chondrocytes of Sprague–Dawley rats injected with saline control (con) or MIA for 1, 2, or 3 weeks. **h** Protein quantification of (**g**) using ImageJ. *n* = 3 per group. **i** Representative images of Safranin O and immunohistochemistry (IHC) staining of PARP12, COL2A1, and MMP13 in chondrocytes of Sprague–Dawley rats. Scale bars: 250 µm (first row) and 50 µm (second row). **j** Quantification of OARSI score based on staining results in (**i**). *n* = 3 per group. **k**–**m** Quantification of PARP12, COL2A1, and MMP13 positive chondrocytes based on staining results in (**i**). *n* = 3 per group. **n** Colocalization of PARP12 and p62 in chondrocytes of Sprague–Dawley rats. Scale bars: 10 µm. **o** Colocalization of PARP12 and mitotracker in chondrocytes of Sprague–Dawley rats. Scale bars: 10 µm. Data are presented as the mean ± SD. One-way analysis of variance with Dunnett’s multiple comparisons test (**c**–**f**, **h**, **k**–**m**) and Kruskal–Wallis test followed by Dunn’s multiple comparisons test (**j**) were used for statistical analysis. *P < 0.05, **P < 0.01, and ***P < 0.001

In recent years, numerous studies have utilized the MIA-induced OA model.^5,41,42^ To further investigate the PARP12 expression pattern, mitophagy process, and cartilage degeneration during OA progression and verify the aforementioned findings in vivo, we established a monosodium iodoacetate (MIA)-induced rat OA model (Fig. 2a). We confirmed the establishment of the rat OA model by 3D reconstruction images of micro-CT scans (Fig. 2b). With the MIA treatment time, the surface roughness, cracks and local defects of condyles of the femur and tibial plateau became more obvious, indicating the degeneration of articular cartilage aggravated. Moreover, we also found that the bone volume /trabecular volume ratio (BV/TV), bone surface/trabecular volume ratio (BS/TV), trabecular thickness, and trabecular numbers of the femur and tibial plateau were significantly decreased in the MIA 2 weeks and 3 weeks groups compared with those in the control (Fig. 2c–f). Furthermore, the relative levels of PARP12 protein were gradually elevated in the MIA-induced OA model and showed statistically significant differences between the 2 weeks and 3 weeks groups (Fig. 2g, h). Consistent with the in vitro assays, IHC staining revealed that the protein levels of PARP12 were gradually increased in the MIA-induced OA model (Fig. 2i, k). Concomitantly, we noticed that the protein levels of COL2A1 and MMP13 were decreased and significantly increased, respectively, in the MIA 2 weeks and 3 weeks groups. Subsequently, we further performed Safranin-O staining, OARSI score evaluations, and IHC analyses of COL2A1 and MMP13 to evaluate cartilage degradation, and the results did not reveal significant differences between the control and the MIA 2 weeks and 3 weeks groups (Fig. 2i, j, l, m). Considering the MIA-induced OA model exhibiting a distinct phenotype of pronounced synovial inflammation, we also conducted an investigation into the PARP12 levels in the synovium. IHC findings suggested that the expression of PARP12 in the synovium increases with prolonged exposure to MIA treatment, and there was a significant difference between the MIA 2 weeks and 3 weeks groups compared to the control group (Fig. S2A, B).

However, autophagic marker LC3B-II/I was downregulated while p62 was upregulated during OA development in MIA-induced rats (Fig. 2h, h). Immunofluorescence of PARP12 and p62 in primary rat chondrocytes also showed that PARP12 colocalized with p62 and mitotracker (Fig. 2n, o). Then, we further confirmed that the co-localization intensity of PARP12 with p62 and mitotracker were also increased in primary rat chondrocytes from MIA 2 weeks group compared with the control group, as indicated by immunofluorescence (Fig. S2C–F). These findings suggested that the impaired mitophagic flux in the MIA-induced rat model changed accordingly with the progression of OA and PARP12 was associated with mitophagy.

### PARP12 regulated mitophagy and degeneration of chondrocytes

**Fig. 3:**
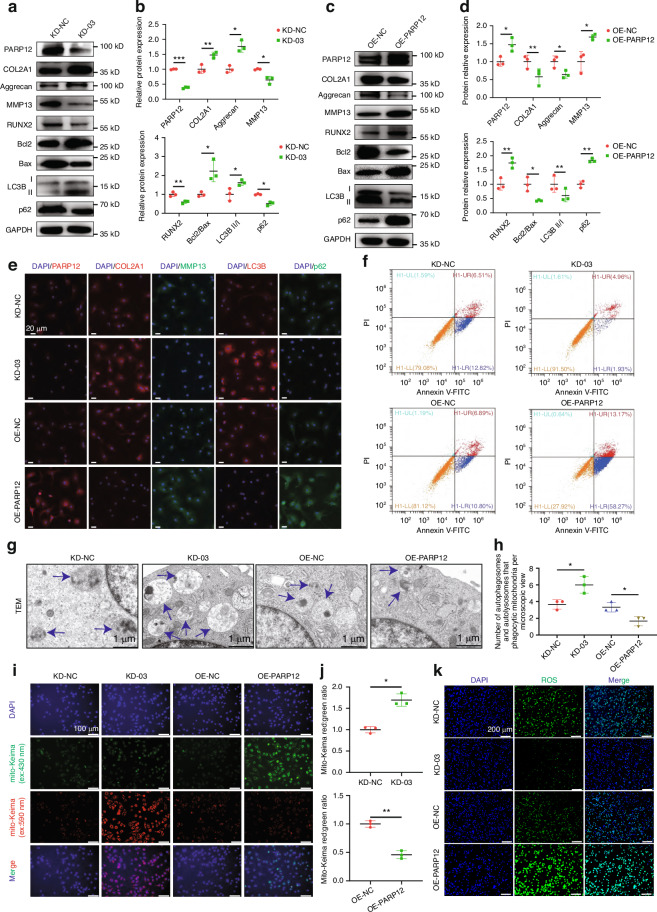
Targeting the expression of PARP12 affects mitophagy and OA-related degeneration in primary human chondrocytes (PHCs). **a** Western blot analysis of COL2A1, aggrecan, MMP13, RUNX2, Bcl2/Bax, LC3B and p62 in PHCs infected with PARP12-overexpressing (OE) or OE-NC adenoviruses. **b** Protein quantification of (**a**) using ImageJ. *n* = 3 per group. **c** Western blot analysis of COL2A1, aggrecan, MMP13, RUNX2, Bcl2/Bax, LC3B and p62 in PHCs infected with *PARP12* KD-03 shRNA or KD-NC shRNA. **d** Protein quantification of (**c**) using ImageJ. *n* = 3 per group. **e** Immunofluorescence of PARP12, COL2A1, MMP13, LC3B and p62 in PHCs following either knockdown or overexpression of PARP12. *n* = 3 per group. Scale bars: 20 µm. **f** Chondrocytes apoptosis was assayed by flow cytometry. **g** Transmission electron microscopy (TEM) of autophagosomes (APs) and autolysosomes (ALs) that phagocytic mitochondria in PHCs following either knockdown or overexpression of PARP12. Blue arrows show APs and ALs that phagocytic mitochondria. Scale bars: 1 µm. **h** Quantification of APs and ALs that phagocytic mitochondria of (**g**). *n* = 3 per group. **i** PHCs were transfected with mito-Keima, incubated with knockdown or overexpression of PARP12 for 48 h, and then observed by fluorescence microscopy. Scale bars: 100 µm. **j** The relative ratio of red to green fluorescence area per cell of (**i**) was quantified. **k** Reactive oxygen species (ROS) staining in PHCs following either knockdown or overexpression of PARP12. Scale bars: 200 µm. Data are presented as the mean ± SD. Paired *t*-test (**b**, **d**, **h**, **j**) was used for statistical analysis. *P < 0.05, **P < 0.01, and ***P < 0.001

To decipher the underlying mechanism of PARP12 in OA pathogenesis, we transfected PHCs with four shRNAs and selected the KD-03 shRNA with the highest knockdown efficiency to carry out subsequent experiments (Fig. S3A). We then assessed the effect of knocking down PARP12 in chondrocytes. Interestingly, PARP12 knockdown significantly upregulated COL2A1, aggrecan, Bcl2/Bax and LC3B (or LC3B-II/I protein) but downregulated MMP13, RUNX2, and p62 in PHCs, as revealed by RT-qPCR (Fig. S3B) and western blot analyses (Fig. 3a, b). Thus, to further investigate the important role of PARP12 in OA development, we transfected PHCs with overexpression-PARP12 (OE-PARP12) or overexpression-NC (OE-NC) adenoviruses and confirmed their efficiency by RT-qPCR (Fig. S3C). As expected, we found that the mRNA and protein levels of COL2A1, aggrecan, Bcl2/Bax and LC3B (or LC3B-II/I protein) were downregulated while those of MMP13, RUNX2, and p62 were upregulated in OE-PARP12 PHCs (Fig. 3c, d and Fig. S3D). Moreover, immunofluorescence further verified that PARP12 affected the expression levels of COL2A1, MMP13, LC3B, and p62 in PHCs (Fig. 3e and Fig. S3E). In addition, enhanced chondrocyte apoptosis was detected in OE-PARP12 group (Fig. 3e and Fig. S3F). These results suggested that PARP12 promotes cartilage degeneration.

We next performed gain-of-function experiments to investigate the important role of PARP12 in the mitophagy of PHCs. Interestingly, transmission electron microscopy (TEM) indicated that the levels of autolysosomes (ALs) and autophagosomes (APs) that phagocytic mitochondria were upregulated after PARP12 knockdown but downregulated after PARP12 overexpression (Fig. 3g, h). Furthermore, mito-Keima detection demonstrated that PARP12 overexpression decreased the number of mitochondria transported to lysosomes (Fig. 3i, j). The colocalization of mitochondria with lysosomes was greater after PARP12 knockdown but downregulated after PARP12 overexpression, which indicates the mitophagy activity being activated in the acidic conditions (Fig. S3G, H). Similarly, evaluation of the intracellular reactive oxygen species (ROS) revealed that the ROS levels were decreased after PARP12 knockdown but upregulated after PARP12 overexpression (Fig. 3k and Fig. S3I). We also found that PARP12 inhibited the ATP production (Fig. S3J). Collectively, the above results demonstrated that PARP12 suppresses the mitophagy of PHCs and is important for the maintenance of cartilage homeostasis.

### PARP12 interacted with ISG15

**Fig. 4:**
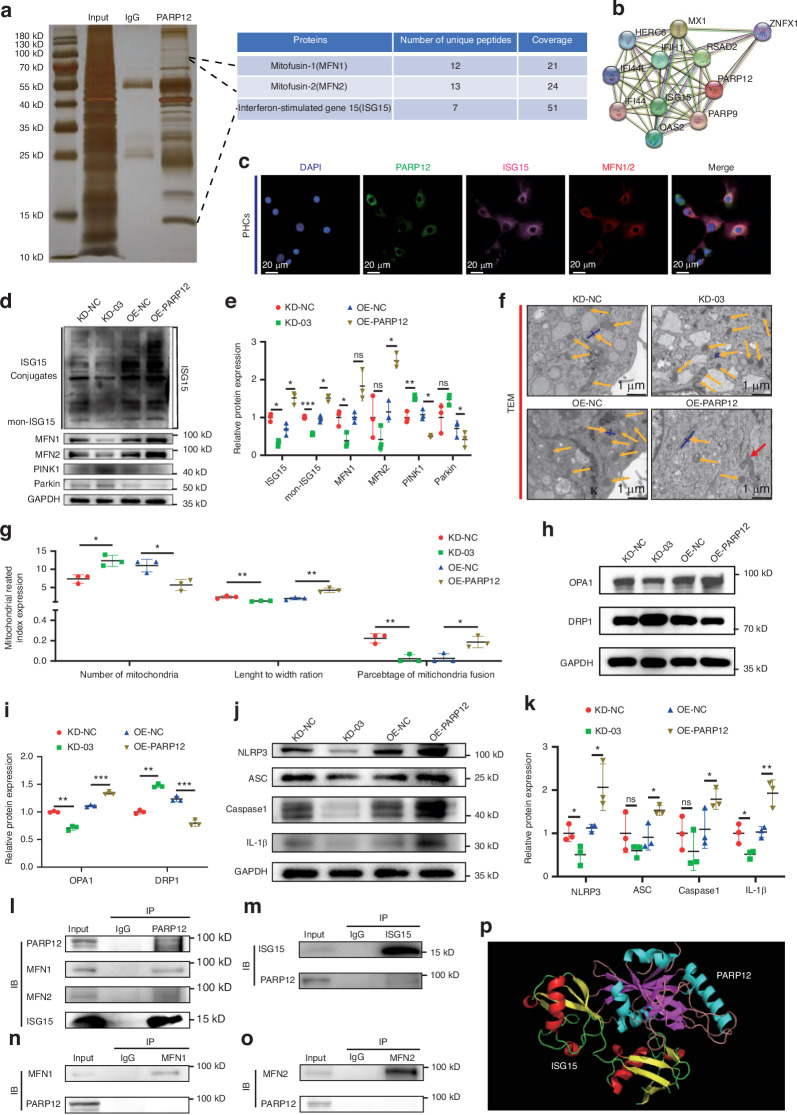
PARP12 interacted with ISG15. **a** Immunoprecipitation-mass spectrometry (IP-MS) analyses of PARP12 interaction proteins. **b** The string database of interaction network diagram between PARP12 and other proteins. **c** Colocalization of PARP12, ISG15, and MFN1/2 in primary human chondrocytes (PHCs). Scale bars: 20 µm. *n* = 3 per group. **d** Western blot analysis of ISG15, MFN1/2, PINK1 and Parkin in PHCs following either knockdown or overexpression of PARP12. **e** Protein quantification of (**d**) using ImageJ. *n* = 3 per group. **f** Representative TEM images of mitochondria in PHCs following either knockdown or overexpression of PARP12. Red arrows show mitochondria fusion. Orange arrows show mitochondria. Blue lines represent the schematic measurement of mitochondrial length and width. Scale bars: 1 µm. **g** Quantification of numbers, length-to-width ratio, and percentage of mitochondria fusion of (**f**). *n* = 3 per group. **h** Western blot analysis of OPA1 and DRP1 in PHCs following either knockdown or overexpression of PARP12. **i** Protein quantification of (**h**) using ImageJ. *n* = 3 per group. **j** Western blot analysis of NLRP3 inflammasome activity in PHCs following either knockdown or overexpression of PARP12. **k** Protein quantification of (**j**) using ImageJ. *n* = 3 per group. **l** Coimmunoprecipitation of PARP12, ISG15, and MFN1/2 in PHCs. *n* = 3 per group. **m** Coimmunoprecipitation of ISG15 and PARP12 in PHCs. *n* = 3 per group. **n** Coimmunoprecipitation of MFN1 and PARP12 in PHCs. *n* = 3 per group. **o** Coimmunoprecipitation of MFN2 and PARP12 in PHCs. *n* = 3 per group. **p** Molecular docking between PARP12 and ISG15. Data are presented as the mean ± SD. Paired *t*-test (**e**, **g**, **i**, **k**) was used for statistical analysis. *P < 0.05, **P < 0.01, and ***P < 0.001

To further explore the mechanism underlying the PARP12-mediated inhibition of mitophagy and promotion of cartilage degeneration in OA, we performed immunoprecipitation (IP) in PHCs and then mass spectrometry to identify potential PARP12 interaction proteins (Fig. 4A). The results showed that among all proteins identified, some were strongly associated with ATP-dependent activity related mitophagy, protein post-translational modifications and the ubiquitin-proteasome pathway (Fig. S4A–C). Among proteins interacting with PARP12, MFN1/2 associated with mitochondrial fusion and mitophagy, attracted our interest. In addition, similar to ubiquitin, interferon-stimulated gene (ISG) 15 is conjugated to cellular proteins (ISGylation) using ISG15-specific ligation enzymes to exert its biological effects through such post-translational modifications and regulate the ubiquitin/26S proteasome.^43,44^ Interestingly, the peptide sequence of the MFN1/2 and ISG15 proteins were detected in the purified complex, and MFN1/2 and ISG15 proteins were not observed among the IgG binding proteins, suggesting that MFN1/2 and ISG15 were bound to PARP12 (Fig. 4a). PARP12 was also shown to interact with ISG15 in the STRING database (Fig. 4b). We further performed immunofluorescence analysis and observed a significant overlap of PARP12, ISG15, and MFN1/2 proteins in PHCs (Fig. 4c).

We then proposed that PARP12 knockdown resulted in downregulation of ISG15 and MFN1/2, whereas that upregulated the expression of PINK1-Parkin which were markers of mediating mitophagy downstream of MFN1/2.^40,45^ Conversely, the expression of ISG15, MFN1/2, and Parkin were changed correspondingly with the overexpression of PARP12 in PHCs (Fig. 4d, e). Next, we further confirmed that PARP12 promoted mitochondrial fusion and inhibited mitophagy in PHCs. TEM analysis revealed that PARP12 downregulated the number of mitochondria, whereas upregulated the length-to-width radio of mitochondria and levels of mitochondrial fusion in PHCs (Fig. 4f, g). Moreover, western blot results of PARP12 knock down and overexpression in PHCs also showed that upon PARP12 knockdown, the mitochondrial fusion marker OPA1 level was downregulated, and the mitochondrial fission marker DRP1 expression was upregulated. Conversely, overexpression of PARP12 led to an increase in OPA1 level and a decrease in DRP1 expression, further confirming that PARP12 could promote mitochondrial fusion in chondrocytes (Fig. 4h, i). Considering that NLRP3 inflammasome mediated pyroptosis of chondrocyte is an important risk factor for the development of OA,^46,47^ and previous studies have also found that mitophagy could delay the progression of inflammatory diseases by inhibiting the formation of the NLRP3 inflammasome.^48,49^ Therefore, this study further explored the impact of PARP12 on the formation of the NLRP3 inflammasome in OA. The results of western blot analysis revealed that PARP12 promoted the activity of the NLRP3 inflammasome (Fig. 4j, k).

Next, we confirmed the interaction between PARP12 and ISG15 or MFN1/2 in PHCs (Fig. 4l–o), unexpectedly, immunoblotting (IB) results indicated the lack of PARP12 when performing IP using an MFN1/2 antibody, which showed that the unstable interaction of PARP12 with MFN1/2. However, we repeatedly verified that PARP12 had a strong interaction with ISG15. Therefore, we explored the mechanism by which PARP12 regulated the levels of MFN1/2 and mitophagy focusing on the downstream target ISG15. Molecular docking was applied to predict the interaction model among PARP12 and ISG15 (Fig. 4p). Taken together, these findings demonstrated that PARP12 interacted with ISG15 and promoted its expression. Next, we explored the mechanism how ISG15 promoted the expression of MFN1/2 and inhibited PINK1/Parkin-mediated mitophagy.

### ISG15 upregulated by PAPR12, increasing ISGylation of MFN1/2 and then attenuating the ubiquitylation and SUMOylation to inhibit PINK1/Parkin-dependent mitophagy

We firstly explored the role of ISG15 in OA. Mitotracker staining revealed an abnormal asymmetric accumulation of stain and increased staining of mitochondria compared with those in PHCs after the knockdown of ISG15 (KD-ISG15) (Fig. S5A, B). ROS were downregulated while JC-1 red/green fluorescence and ATP were upregulated in KD-ISG15 PHCs (Fig. S5C–G). In addition, the protein levels of LC3B-II/I, PINK1 and Parkin were upregulated while those of MFN1/2 and p62 were downregulated after KD-ISG15 (Fig. S5H, I). These results showed that ISG15 inhibited PINK1/Parkin-mediated mitophagy in OA.

**Fig. 5:**
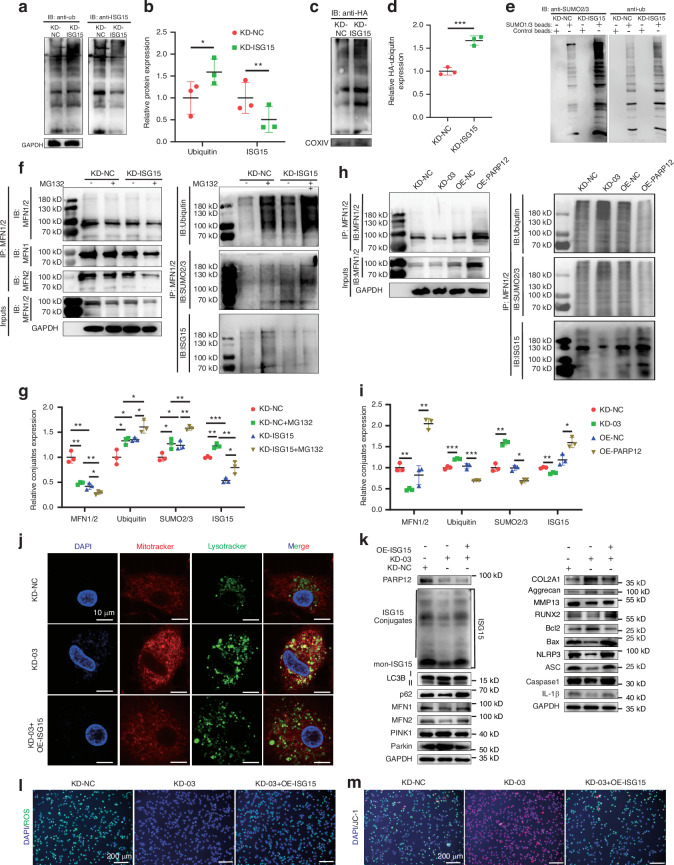
ISG15 upregulated by PAPR12, increasing ISGylation of MFN1/2 and then attenuating the ubiquitylation and SUMOylation to inhibit PINK1/Parkin-dependent mitophagy. **a** Immunoblotting (IB) analysis of ubiquitylation and ISG15 in PHCs following transfection with KD-ISG15 or KD-NC. **b** Protein quantification of (**a**) using ImageJ. *n* = 3 per group. **c** IB analysis of ubiquitylation of mitochondrial proteins. Mitochondria were isolated from KD-NC and KD-ISG15 PHCs transfected with a HA-ubiquitin construct. **d** Protein quantification of (**d**) using ImageJ. *n* = 3 per group. **e** IB analysis of SUMO2/3 and ubiquitin with SUMO2/3-conjugated proteins in KD-NC and KD-ISG15 PHCs immunoprecipitated using SUMO2/3 affinity beads. **f** IB analysis of MFN1/2, MFN1, MFN2, ubiquitin, SUMO2/3 and ISG15 in KD-NC and KD-ISG15 PHCs treated with or without MG-132 after immunoprecipitation of MFN1/2. The molecular marker of IB plots of ubiquitin, SUMO2/3, and ISG15 conjugates with MFN1/2 is 70 kD and above when considering the molecular weight of MFN1/2 at 85 kD. **g** Quantification of MFN1/2, ubiquitin, SUMO2/3, and ISG15 of (**f**) using ImageJ after immunoprecipitation of MFN1/2. *n* = 3 per group. **h** IB analysis of MFN1/2, MFN1, MFN2, ubiquitin, SUMO2/3, and ISG15 in PHCs following either knockdown or overexpression of PARP12 after immunoprecipitation of MFN1/2. **i** Quantification of MFN1/2, ubiquitin, SUMO2/3, and ISG15 of (**h**) using ImageJ. *n* = 3 per group. **j** MitoTracker Red and LysoTracker Green staining of PHCs was observed by confocal microscopy. Scale bars: 10 µm. **k** Western blot analysis of PARP12, ISG15, LC3B, p62, MFN1, MFN2, PINK1, Parkin, COL2A1, aggrecan, MMP13, RUNX2, Bcl2, Bax, and NLRP3 inflammasome activity in PHCs coinfected with *PARP12* KD-03 shRNA and ISG15-OE adenovirus. *n* = 3 per group. **l** ROS staining with PARP12 knockdown and ISG15 overexpression. Scale bars: 100 µm. **m** JC-1 staining in PHCs following transfection with PARP12 knockdown and ISG15 overexpression. Scale bars: 100 µm. Data are presented as the mean ± SD. Paired *t*-test (**b**, **d**, **i**) and one-way analysis of variance followed by Tukey’s multiple comparison test (**g**) were used for statistical analysis. *P < 0.05, **P < 0.01, and ***P < 0.001

Then, we observed that the fluorescence intensity of small ubiquitin-like modifier (SUMO)-2/3 and its colocalization with MFN1/2 were increased in KD-ISG15 PHCs, as indicated by immunofluorescence (Fig. S5J–L). Consistently, we also detected that the level of ubiquitin was upregulated in KD-ISG15 PHCs (Fig. 5a, b). We further evaluated the level of ubiquitination of mitochondrial proteins following mitochondria isolation and found that it was also elevated after KD-ISG15 (Fig. 5c, d). Finally, using SUMO2/3 affinity beads, we detected that the levels of SUMOylation and ubiquitylation were increased in KD-ISG15 PHCs (Fig. 5e and Fig. S5M). These results indicated that ISG15 inhibited ubiquitylation and SUMOylation of mitochondrial proteins in PHCs.

We further applied the ubiquitination inhibitor TAK-243^50^ and the SUMOylation inhibitor ML-792^51^ to PHCs to evaluate the function of ubiquitination and SUMOylation in OA. Western blot analysis revealed that TAK-243 and ML-792 treatment significantly reduced ubiquitin and SUMO2/3-conjugate levels in a concentration-dependent manner (Fig. S6 A, B, F, G). Furthermore, TAK-243 and ML-792 at concentrations greater than or equal to 50 nmol/L both significantly downregulated the protein levels of COL2A1 and Bcl2/Bax, while upregulated the protein level of MMP13. The results of CCK-8 assay revealed that treatment of chondrocytes with 300 nmol/L concentrations of TAK-243 and ML-792 induced a significant inhibition of cell viability compared to the control group (Fig. S6C, H). Therefore, we further applied TAK-243 and ML-792 at concentrations of 50 nmol/L and 100 nmol/L to chondrocytes, respectively. Immunofluorescence results confirmed that both TAK-243 and ML-792 significantly reduced the protein levels of COL2A1, while promoting the expression of MMP13 (Fig. S6D, E, I, J). Overall, these results indicated that the ubiquitination inhibitor TAK-243 and the SUMOylation inhibitor ML-792 promoted cartilage degeneration and OA progression by inhibiting the ubiquitination and SUMOylation levels in chondrocytes. These also suggested that the functions of ubiquitination and SUMOylation in chondrocytes are to inhibit the OA progression.

We next examined the fate of MFN1/2 and associated post-translational modifications (ubiquitylation, SUMOylation, and ISGylation) in PHCs transfected with KD-ISG15 or KD-NC in the presence or absence of the proteasome inhibitor MG132. Our IP results showed that the levels of MFN1/2 were decreased while those of their post-translational modifications (ubiquitylation, SUMOylation, and ISGylation) were increased after applying MG132. Compared with the findings in KD-NC PHCs, the MFN1/2 levels were downregulated while ubiquitylation and SUMOylation were upregulated in KD-ISG15 PHCs with decreasing ISGylation (Fig. 5f, g).

Based on our previous findings that PARP12 interacts with ISG15 and promotes its expression (Fig. 4d, e), we further investigated the role of PARP12 in regulating MFN1/2 and associated post-translational modifications. The results showed that MFN1/2 and its ISGylation were downregulated while ubiquitylation and SUMOylation were upregulated in PHCs after PARP12 knockdown. In contrast, MFN1/2 and its ISGylation were upregulated while ubiquitylation and SUMOylation of MFN1/2 were downregulated in PHCs after PARP12 overexpression (Fig. 5h, i). Finally, we further explored the interaction between PARP12 and ISG15 in regulating mitophagy and cartilage degeneration. Functional rescue experiments confirmed that PAPR12 inhibited mitophagy and promoted the activation of the NLRP3 inflammasome by upregulating ISG15 to significantly accelerate cartilage degeneration, which was also revealed by mitoTracker Red and lysoTracker green staining, mito-Keima, western blotting, ROS and JC-1 staining (Fig. 5j–m and Fig. S7A–L). Together, these data demonstrated that ISG15 was upregulated by PAPR12, increasing ISGylation of MFN1/2 and then attenuating the ubiquitylation and SUMOylation to upregulate MFN1/2 levels, which inhibited the PINK1/Parkin-mediated mitophagy in OA.

### Inflammatory cytokine-induced IRF1 activation promoted PARP12 transcription

**Fig. 6:**
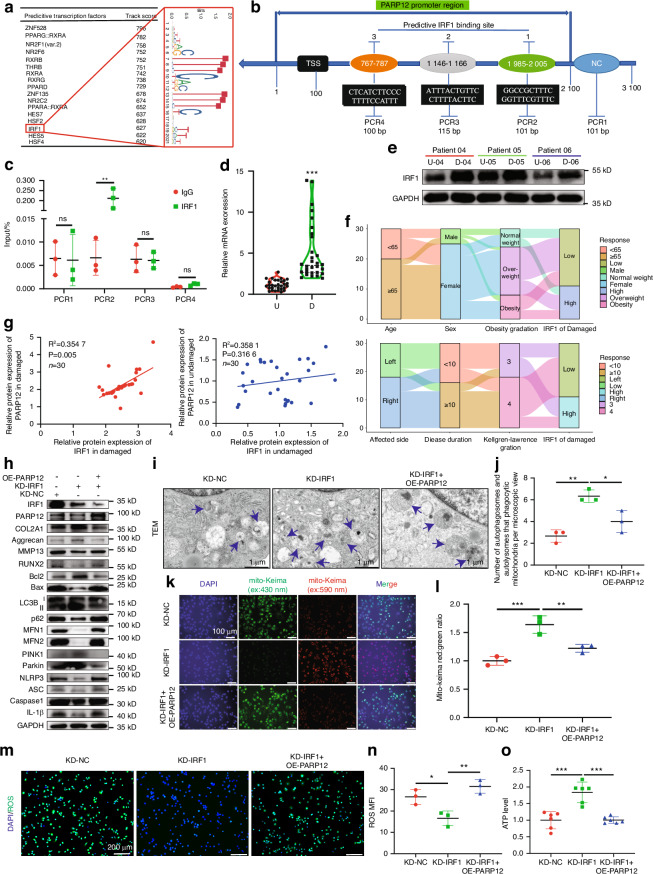
Inflammatory cytokines-induced IRF1 activation promoted PARP12 transcription. **a** The JASPAR database was used to predict potential transcription factors (TFs) of PARP12 (*H. sapiens*). **b** Schematic of the predictive binding site of IRF1 at the PARP12 promoter region 2 000-bp upstream of the transcription start site (TSS), with the TSS being designated as 100. A 2 100–3 100 bp fragment was used as the negative control (NC). **c** Quantification of immunoprecipitated DNA by PCR following a chromatin immunoprecipitation (ChIP) assay in PHCs using normal rabbit IgG as the NC. **d** Quantitative PCR analysis of IRF1 in U and D cartilage tissues. *n* = 30 per group. **e** Western blot analysis of IRF1 between U and D cartilage tissues. **f** Relationship between age, sex, obesity gradation, affected side, disease duration, Kellgren–Lawrence gradation, and expression of IRF1 in the damaged area represented by the Sankey diagram. **g** Linear regression analysis of the expression of IRF1 and PARP12 in U and D areas in patients with knee OA (*n* = 30). **h** Western blot analysis of IRF1, PARP12, COL2A1, aggrecan, MMP13, RUNX2, Bcl2, Bax, LC3B, p62, MFN1, MFN2, PINK1, Parkin and NLRP3 inflammasome activity in PHCs coinfected with *IRF1* KD shRNA and PARP12-OE adenovirus. *n* = 3 per group. **i** TEM of APs and ALs that phagocytic mitochondria. Blue arrows show APs and ALs that phagocytic mitochondria. Scale bars: 1 µm. **j** Quantification of APs and ALs that phagocytic mitochondria of (**i**). *n* = 3 per group. **k** The represent images of PHCs transfected with mito-Keima, coinfected with *IRF1* KD shRNA and PARP12-OE adenovirus. *n* = 3 per group. Scale bars: 100 µm. **l** The relative ratio of red to green fluorescence area per cell of (**k**) was quantified. **m** ROS staining. Scale bars: 200 µm. **n** ROS quantification of (**m**) via ImageJ. *n* = 3 per group. **O** Quantification of the ATP level. *n* = 6 per group. Data are presented as the mean ± SD. Paired *t*-test (**c**, **d**), simple linear regression (**g**), and one-way analysis of variance followed by Tukey’s multiple comparison test (**j**, **l**, **n**, **o**) were used for statistical analysis. *P < 0.05, **P < 0.01, and ***P < 0.001

As both PARP12 mRNA and protein levels were increased in D areas of knee OA cartilage and PHCs stimulated with IL-1β, we proposed that PARP12 expression was under transcriptional regulation. Thus, to examine the mechanisms underlying the upregulation of the expression of PARP12, we first predicted 17 potential transcription factors (TFs) of PARP12 (*Homo sapiens*) using the JASPAR database (Fig. 6a). Among them, the expression of IRF1was significantly upregulated in the D cartilage samples in our mRNA-seq analyses (10.07-fold). The GEPIA database also indicated that the expression of PARP12 was positively correlated with IRF1 (Fig. S8A). To verify whether IRF1 directly targets the promoter region of PARP12, we performed chromatin IP (ChIP) experiments and RT-qPCR. The ChIP analysis revealed that a direct target occurred between IRF1 and the PARP12 promoter at the predictive binding site 1 (Fig. 6b, c). We next found that the mRNA and protein levels of IRF1 were increased in the D cartilage of 30 patients with knee OA (same samples used for testing of PARP12) by RT-qPCR (Fig. 6d, e and Fig. S8B). The relationship between patient characteristics and IRF1 expression in the D area of the cartilage were shown in a Sankey diagram (Fig. 6f), which indicated that IRF1 was associated with age, sex and Kellgren-Lawrence gradation. Interestingly, we found that IRF1 was significantly and positively correlated with the level of PARP12 in the D cartilage (*P* = 0.005); however, these correlations were not observed in the U cartilage (Fig. 6g).

Next, we further explored the relationship between IRF1 and PARP12, ISG15, MFN1/2, and mitophagy. Western blot results indicated that IRF1 knockdown significantly downregulated PARP12, ISG15, MFN1/2 and p62 but upregulated PINK1, Parkin and LC3B-II/I in PHCs, while PARP12, ISG15, MFN1/2 and p62 levels were increased but PINK1, Parkin and LC3B-II/I expression were decreased after IRF1 overexpression (Fig. S8C, D). Furthermore, mito-Keima detection demonstrated that IRF1 knockdown increased the number of mitochondria transported to lysosomes, while the number of mitochondria transported to lysosomes were upregulated after IRF1 overexpression, which indicates the mitophagy activity being activated in the acidic conditions (Fig. S8E, F). Moreover, evaluation of the intracellular ROS revealed that the ROS levels were decreased after IRF1 knockdown but upregulated after IRF1 overexpression (Fig. S8G, H). Collectively, the above results demonstrated that IRF1 promotes PARP12, ISG15, MFN1/2 levels and suppresses the mitophagy of PHCs.

Western blots, mito-Keima staining, ROS staining, JC-1 staining and ATP detection were performed, and the results showed that IRF1 inhibited PINK1/Parkin-mediated mitophagy and promoted cartilage degeneration, which could be significantly reversed by PARP12 (Fig. 6h–o and Fig. S9A–I). Overall, these data indicated that inflammatory cytokine-induced IRF1 activation promoted PARP12 transcription via binding to PARP12 promoter.

### XAV-939 promoted PINK1/Parkin-mediated mitophagy and suppressed cartilage degradation via targeting PARP12

**Fig. 7:**
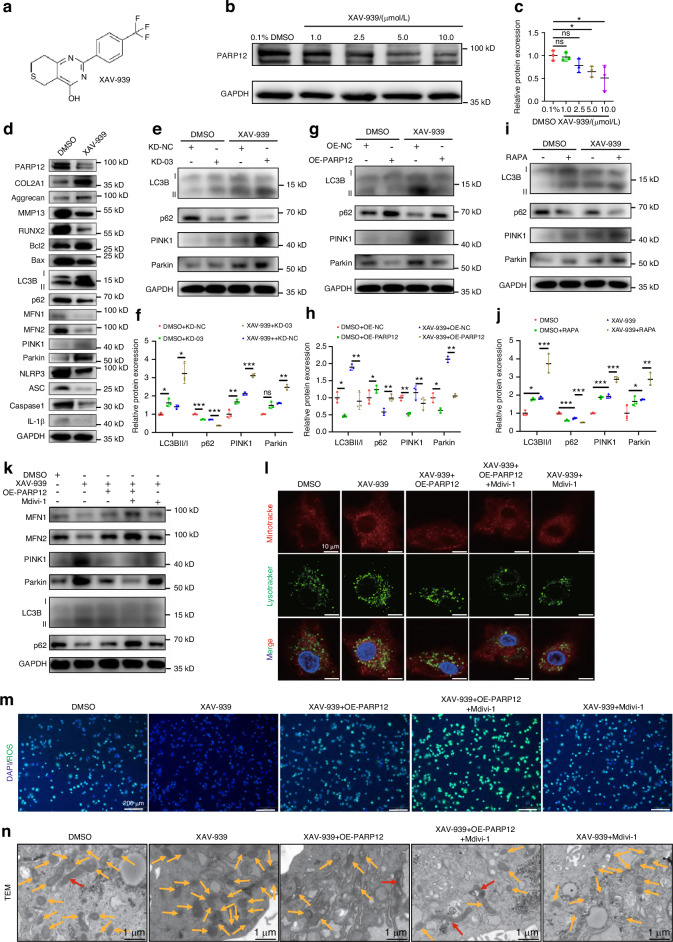
XAV-939 promoted PINK1/Parkin-mediated mitophagy and suppressed cartilage degradation via targeting PARP12. **a** Chemical formula structure of XAV-939. **b** Western blot analysis of PARP12 in primary human chondrocytes (PHCs) treated with different concentrations of XAV-939. **c** Protein quantification of (**b**) using ImageJ. *n* = 3 per group. **d** Western blot analysis of PARP12, COL2A1, aggrecan, MMP13, RUNX2, Bcl2/Bax, LC3B, p62, MFN1, MFN2, PINK1, Parkin and NLRP3 inflammasome activity in PHCs treated with XAV-939. **e** Western blot analysis of LC3B, p62, PINK1 and Parkin in PHCs treated with XAV-939 or DMSO and transfected with *PARP12* KD-03 shRNA or NC. **f** Protein quantification of (**e**) using ImageJ. *n* = 3 per group. **g** Western blot analysis of LC3B, p62, PINK1 and Parkin in PHCs treated with XAV-939 or DMSO and transfected with PARP12-OE adenovirus or NC. **h** Protein quantification of (**g**) using ImageJ. *n* = 3 per group. **i** Pretreatment with RAPA (1 μmol/L) for 1 h significantly changed the expression of LC3B, p62, PINK1 and Parkin treated by XAV-939 in PHCs. **j** Protein quantification of (**i**) using ImageJ. *n* = 3 per group. **k**–**m** Western blot analysis, fluorescence analysis of MitoTracker Red and LysoTracker Green staining and ROS staining to detect mitophagic flux of PARP12 and XAV-939. **n** Representative TEM images of mitochondria in PHCs. Red arrows show mitochondria fusion. Orange arrows show mitochondria. Scale bars: 1 µm. Data are presented as the mean ± SD. One-way analysis of variance with Dunnett’s multiple comparisons test (**c**, **j**) and paired *t*-test (**f**, **h**) were used for statistical analysis. *P < 0.05, **P < 0.01, and ***P < 0.001

A previous study found that within the PARP family, XAV-939 has a pronounced inhibitory effect on PARP12, while it is not sensitive to other PARPs.^52^ Therefore, this study selected XAV-939 for investigation. Western blot analysis revealed that 5 μmol/L and 10 μmol/L XAV-939 were effective in inhibiting the expression of PAPR12 in PHCs compared with the control (Fig. 7b, c). Following the principle of effective and economical dose of drug therapy, the 5 μmol/L dose was applied in all following experiments. In addition, the results of western blots indicated that there was no significant difference in IRF1 levels of between the XAV-939 treated groups and the control group (Fig. S10A, B). In summary, XAV-939 directly inhibited the expression of PARP12 rather than suppressing PARP12 levels through the inhibition of IRF1 levels.

We observed that the mRNA and protein levels of COL2A1 and aggrecan were significantly upregulated while those of MMP13 and RUNX2 were downregulated in PHCs treated with XAV-939 (Fig. 7d and Fig. S10C, D). In addition, XAV-939 significantly upregulated the protein levels of PINK1/Parkin-dependent mitophagy but downregulated the activity of the NLRP3 inflammasome and cartilage degeneration. Immunofluorescence confirmed that the levels of PARP12, MMP13, and p62 were decreased while those of COL2A1 and LC3B increased in XAV-939-treated PHCs (Fig. S10E, F). We next performed JC-1 staining and ATP detection identified that XAV-939 promoted mitophagy (Fig. S10G–I). Transfection of cells with shRNA-PARP12 further increased XAV-939-treated mitophagy, as determined by the levels of LC3B-II/I, p62, PINK1 and Parkin in PHCs (Fig. 7e, f). XAV-939 application also reversed the levels of mitophagy in PHCs with PARP12 overexpression (Fig. 7g, h). To further explored the role of XAV-939 in mitophagy, we treated PHCs with rapamycin (RAPA, 1 μmol/L) to induce mitophagy: RAPA significantly further increased XAV-939-induced the level of PINK1/Parkin-dependent mitophagy (Fig. 7i, j).

To assess the effects of PARP12 and XAV-939 on mitophagic flux, PHCs were treated with OE-PARP12 adenoviruses, XAV-939 or Mdivi-1 (10 μmol/L), a mitophagy inhibitor. Western blotting, the overlapped signal between mitoTracker Red and lysoTracker green staining and ROS staining were performed. As shown in Fig. 7k–m and Fig. S10J–L, XAV-939 treatment significantly increased the level of mitophagy. Transfecting cells with OE-PARP12 significantly decreased mitophagic flux after XAV-939, which was similar to Midivi-1 treatment. Besides, co-incubation of cells with OE-PARP12 and Mdivi-1 led to decreased the level of mitophagy after XAV-939 compared with OE-PARP12 or Mdivi-1 treatment alone. In addition, TEM analysis revealed that XAV-939 upregulated the number of mitochondria, whereas downregulated the length-to-width radio of mitochondria and levels of mitochondrial fusion in PHCs (Fig. 7n and Fig. S10M). Taken together, these results demonstrated that XAV-939 promoted PINK1/Parkin-mediated mitophagy via targeting PARP12.

The aforementioned studies demonstrated that XAV-939 inhibited cartilage degeneration and the OA progression by suppressing the Wnt signaling pathway.^53,54^ Therefore, we also explored whether XAV-939 inhibited cartilage degeneration not only by inhibiting the Wnt signaling pathway and promoting the expression of Axin2, but also through the target of PARP12. We conducted additional experiments in which PHCs were treated with XAV-939, OE-Wnt3a adenoviruses, shRNA-Axin2 and OE-PARP12 adenoviruses, followed by protein extraction for western blot analysis. Western blot results indicated that in addition to its ability to inhibit the Wnt signaling pathway and promote Axin2 expression, XAV-939 could also significantly suppress the PARP12 levels. We found that treatment with XAV-939 in combination with Wnt3a overexpression or Axin2 knockdown, compared to the control group, could not completely rescue the phenotype of cartilage degeneration. However, further PARP12 overexpression on this basis could further rescue the phenotype of cartilage degeneration, which indicated that in addition to its ability to inhibit cartilage degeneration by suppressing the Wnt signaling pathway or promoting Axin2 levels, XAV-939 could indeed delay the degeneration of cartilage and the OA progression by targeting the inhibition of PARP12 (Fig. S11A–G). In summary, this study uncovered a novel mechanism of action for XAV-939, in addition to its previous roles as a Wnt signaling pathway inhibitor and an Axin2 stabilizer, where it targets PARP12 to regulate cartilage degeneration in OA.

### PARP12 modulated osteoarthritis pathogenesis in rats

**Fig. 8:**
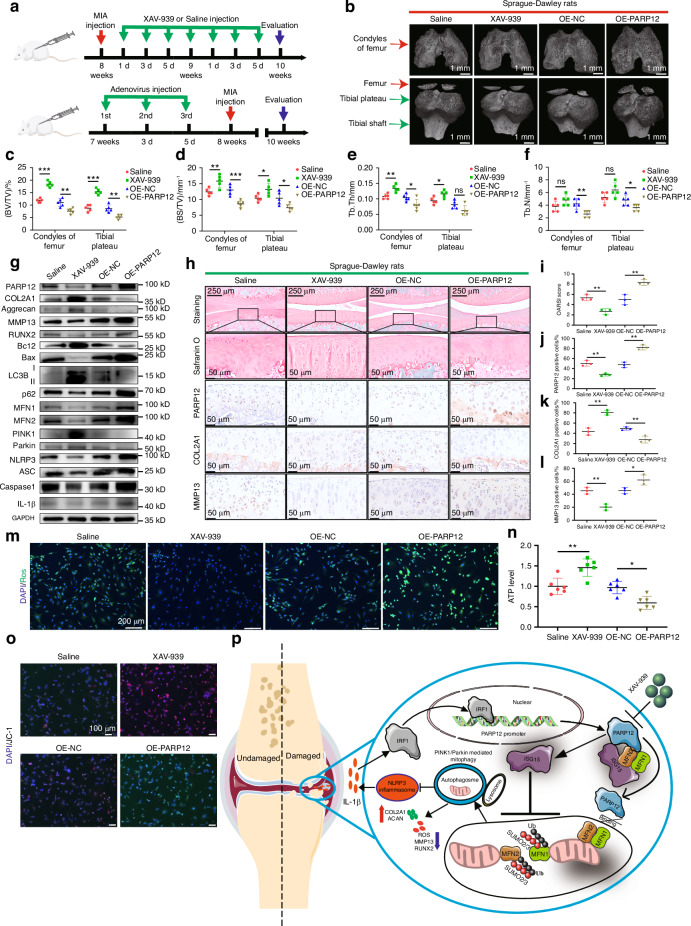
PARP12 modulates osteoarthritis (OA) pathogenesis in monosodium iodoacetate (MIA)-treated rats. **a** Experimental diagram of the MIA OA rat model treated with XAV-939 or PARP12 overexpression (OE) adenovirus. Rats were evaluated at age of 10 weeks. *n* = 6 per group. **b** 3D reconstruction images of micro-CT scanning of the knees of rats treated with XAV-939 or PARP12-OE adenovirus. *n* = 5 per group. **c**–**f** Analysis of BV/TV, BS/TV, trabecular thickness, and trabecular numbers. *n* = 5 per group. **g** Western blot analysis of PARP12, COL2A1, aggrecan, MMP13, RUNX2, Bcl2/Bax, LC3B, p62, MFN1, MFN2, PINK1, Parkin and NLRP3 inflammasome activity in chondrocytes of rats treated with XAV-939 or PARP12 overexpression (OE) adenovirus. *n* = 3 per group. **h** Representative images of Safranin O and IHC staining of PARP12, COL2A1 and MMP13. Scale bars: 250 µm (first row) and 50 µm (second row). **i** Quantification of macroscopic score based on staining results in (**h**). *n* = 3 per group. **j**–**l** Quantification of PARP12, COL2A1, and MMP13 positive chondrocytes based on staining results in (**h**). *n* = 3 per group. **m**–**o** ROS staining, ATP level and JC-1 staining in chondrocytes of rats treated with XAV-939 or PARP12 OE adenovirus. **p** Schematic representation of the mechanism by which IRF1-mediated upregulation of PARP12 promotes cartilage degradation by inhibiting PINK1/Parkin dependent mitophagy through ISG15 attenuating the ubiquitylation and SUMOylation of MFN1/2. Data are presented as the mean ± SD. Paired *t*-test (**c**–**f**, **j**–**l**, **n**) and non-parametric Mann-Whitney U test (**i**) were used for statistical analysis. *P < 0.05, **P < 0.01, ***P < 0.001

To corroborate the above-mentioned findings, we further assessed the effects of PARP12 on OA rats, which were divided into four groups: saline group, XAV-939 group, OE-NC adenovirus group, and OE-PARP12 adenovirus group (Fig. 8a). A 3D reconstruction of the micro-CT scans of rat OA knees revealed that surface roughening, fibrillation, fissures, and erosions down to the subchondral bone were decreased in the XAV-939 group compared with the saline group while all these indicators were increased in the OE-PARP12 adenovirus group compared with the OE-NC adenovirus group (Fig. 8b). Similarly, the BV/TV, BS/TV, trabecular thickness, and trabecular numbers of the femur and tibial plateau were upregulated in the XAV-939 group compared with the saline group but downregulated in the OE-PARP12 adenovirus group compared with the OE-NC adenovirus group (Fig. 8c–f).

Western blot analyses revealed that more severe cartilage degeneration was found in the OE-PARP12 group. Conversely, OA improved in the XAV-939 group (Fig. 8g and Fig. S12A). Therefore, we also performed Safranin-O staining and IHC analysis of PARP12, COL2A1, and MMP13 to evaluate cartilage degradation. The results showed that intraarticular injection of XAV-939 relieved the degenerative changes in the cartilage of rats with MIA-induced OA, which exhibited lower OARSI scores; however, these changes became more severe after overexpression of PARP12 (Fig. 8h–l). Furthermore, the results indicated that intra-articular administration of XAV-939 downregulated the PARP12 levels in the synovium compared with the saline group, whereas increased in the OE-PARP12 adenovirus group compared with the OE-NC adenovirus group (Fig. S12B, C).

Next, we performed ROS and JC-1 staining, and ATP detection to evaluate the levels of mitophagy in chondrocytes of rats in the four treatment groups. Overexpression of PARP12 inhibited mitophagy in MIA-induced OA rats, whereas the opposite effects were observed after XAV-939 treatment (Fig. 8m–o and Fig. S12D, E). Based on these results, we proposed that the IRF1-mediated upregulation of PARP12 promotes cartilage degradation by inhibiting PINK1/Parkin dependent mitophagy through ISG15-based attenuation of the ubiquitylation and SUMOylation of MFN1/2 (Fig. 8p), which represents a novel mechanism for the pathogenesis of OA.

## Discussion

OA is a degenerative joint disease characterized by the activation of chondrocytes by inflammatory cytokines, such as IL-1β, which results in catabolism exceeding anabolism and degradation of the ECM, thereby further promoting OA progression.^55–57^ Mitochondria, the ‘energy powerhouses of the cell’, are critical in maintaining cellular energy homeostasis and involved in various vital cellular processes.^58^ However, mitochondrial dysfunction not only causes ECM degradation but also contributes to inflammasome activation.^59,60^ Mitophagy can effectively remove D mitochondria, thereby inhibiting the activity of the inflammasome and cartilage degeneration.^14,48^ In our previous study, we demonstrated that mitochonic acid-5 inhibited cartilage degeneration by improving Parkin-dependent mitophagy.^61^ However, the underlying mechanism regulating mitophagy during OA progression remains largely unknown.

Recently, PARP12 was reported to be associated with mitochondria in thermogenic adipocytes, but the specific function and mechanism have not been elucidated.^24^ In this study, we identified differentially expressed PARPs by sequencing and confirmed that only the expression of stable PARP12 was significantly different in OA. Furthermore, we examined the expression pattern of PARP12 in a larger number of clinical samples from patients with OA and found that PARP12 was correlated with certain clinical data, especially Kellgren-Lawrence gradation, suggesting that PARP12 may be used to predict the severity of OA. As expected, our findings verified that PARP12 was indeed highly expressed in chondrocytes and cartilage from rats with OA. Meanwhile, we further confirmed that PAPR12 inhibited autophagy after colocalization with p62 in both human and rat chondrocytes, in which catabolism was upregulated and anabolism was downregulated. Additionally, we then elucidated the molecular mechanism underlying the involvement of PARP12 in the regulation of cartilage degeneration. Using mass spectrometry and co-IP assays, we found that ISG15 and MFN1/2 were the binding proteins of PARP12. We further confirmed that PARP12 downregulated the expression of ISG15 and MFN1/2, as well as mitophagy. However, PARP12 did not directly bind to MFN1/2. Thus, we proposed that PARP12 regulates the levels of MFN1/2 and mitophagy via ISG15.

ISG15 and ISGylation in protein post-translational modification, have attracted the increased attention of researchers in various diseases.^44,62^ For example, the ISGylation of skeletal protein Emerin was shown to inhibit ubiquitination and promote aerobic oxidation, thereby stimulating glycolysis in lung adenocarcinoma^63^; moreover, ISG15 promoted mitochondrial ISGylation concomitant with decreases in the accumulation of dysfunctional mitochondria and impairment of mitophagy in pancreatic cancer stem cells.^33^ However, the molecular functions and mechanisms of ISG15 in cartilage degradation remain unclear. We first confirmed that ISG15 can inhibit mitophagy in cartilage through the promotion of ISGylation and attenuation of ubiquitylation and SUMOylation of MFN1/2. In addition, we demonstrated that MFN1/2 were degraded by SUMOylation and ubiquitylation in PHCs while ISGylation inhibited this process, which upregulated the level of Parkin-dependent mitophagy. We further verified that PARP12 inhibited PINK1/Parkin-dependent mitophagy by inhibiting the ubiquitination and SUMOylation of MFN1/2 via upregulating ISG15 expression, which explained the regulation of MFN1/2 without direct interaction with PARP12. SUMOylation is also a ubiquitin-like modification that targets post-translational modifications, and it represents a research hotspot in recent years. A recent study reported the stabilization of lactate through the SUMOylation of two residues on anaphase-promoting complex.^64^ SUMOylation targeting mitophagy by regulating the conjugation of MFN1/2 SERCA2a, HIF1α, and PINK1 has also been reported in cardiovascular diseases.^65^ In this study, we first reported the mechanism of SUMOylation in OA and found that MFN1/2 were not only modified by ubiquitination but also SUMOylated by SUMO2/3, which indicated that MFN1/2 were also degraded by SUMOylation and thus enhanced the level of mitophagy in OA. We further found that upregulation of MFN1/2 inhibited mitophagy and promoted the progression of OA, which is consistent with a previous study.^37^

To explore the regulation of PARP12 in OA, we used the JASPAR database to predict the binding of several TFs in its promoter region (*H. sapiens*).^66^ Among the predicted TF candidates, we selected IRF1 as the predicted potential upstream TF of PARP12, which has also been reported to upregulate catabolism, such as by activating MMP13 in a recent study.^67^ We first showed that IRF1 binds to the promoter region of PARP12. Subsequently, we found that PARP12 was upregulated and positively correlated with the expression of IRF in the D cartilage of patients. Thus, we speculated that IRF1 may regulate cartilage degeneration by targeting PARP12. Consistent with the above assumption, we first demonstrated that inflammatory cytokine-induced IRF1 activation promoted PARP12 transcription via binding to PARP12 promoter.

Various PARP inhibitors have been developed for cancer treatment, and some have been approved by the Food and Drug Administration over the past decades. These inhibitors mainly target PARP1 and PARP2. However, inhibitors targeting other members of the PARP family are less understood.^68^ We found that the XAV-939 inhibitor of PARP12 promoted mitophagy and suppressed the activity of the NLRP3 inflammasome and cartilage degeneration in vitro and in vivo. XAV-939, as a small molecule inhibitor, selectively inhibits Wnt signaling pathway and stabilizes an Axin2 protein, thereby suppressing the occurrence and progression of diseases.^69–71^ In OA, XAV-939 was also found to suppress the onset and progression of the disease by inhibiting the Wnt/β-catenin signaling pathway. For instance, in a murine OA model, the application of XAV-939 inhibited the proliferation and inflammation of synovial fibroblasts and promoted the anabolic metabolism of chondrocytes.^53^ Moreover, intra-articular injection of XAV-939 in the destabilization of the medial meniscus (DMM) murine model displayed a significant alleviation of OA progression.^54^ The aforementioned studies demonstrated that XAV-939 could indeed inhibit cartilage degeneration and the OA progression by suppressing the Wnt signaling pathway, which may have introduced a certain bias to the results of this experiment. To address this limitation, we conducted experiments demonstrated that in addition to XAV-939 inhibit cartilage degeneration by suppressing the Wnt signaling pathway or promoting Axin2 levels, XAV-939 could indeed delay the degeneration of cartilage and the OA progression by targeting the inhibition of PARP12. In this study, we first found XAV-939 could promote mitophagy and inhibit OA progression by targeting PARP12, which is of great significance for developing PARP family inhibitors and expanding the potential targets for XAV-939. More importantly, this study provides new and strong evidence to support XAV-939 as a treatment of OA.

In summary, we first demonstrated that PARP12 inhibits mitophagy and promotes cartilage degeneration. Mechanistically, PARP12 inhibited PINK1/Parkin-dependent mitophagy by inhibiting the ubiquitination and SUMOylation of MFN1/2 by upregulating ISG15 and ISGylation. Furthermore, we also first demonstrated that inflammatory cytokine-induced IRF1 activation promoted PARP12 transcription by binding to the PARP12 promoter. In translational applications, PARP12 can be used to predict the severity of cartilage degeneration; thus, it represents a new target for the study of mitophagy and OA progression; moreover, XAV-939 effectively promotes mitophagy and inhibits cartilage degeneration in OA both in vitro and in vivo by targeting PARP12, which is important for the developing PARP family inhibitors and expanding potential targets for XAV-939. In addition, these findings provide new and strong evidence to support XAV-939 as a treatment of OA.

## Materials and Methods

### Human cartilage and clinical characteristics

The collection of human cartilage samples and relevant clinical characteristics was approved by the Clinical Research Ethics Committee of the First Affiliated Hospital of Sun Yat-sen University ([2021]334). Informed consent was obtained from all participants. Articular cartilage samples were divided into the undamaged (ICRS = 0) and damaged (ICRS = 1-4) groups according to evaluate the International Cartilage Repair Society (ICRS) score.^72^ We collected patient characteristics, including radiography (preoperation and postoperation), age, sex, BMI, obesity gradation (Underweight: BMI < 18.5; Normal weight: 18.5 ≤ BMI < 24; Overweight: 24 ≤ BMI < 28; Obesity: BMI ≥ 28), affected side, disease duration, and Kellgren-Lawrence gradation (upon preoperative radiography).

### Chondrocyte culture

Primary human chondrocytes (PHCs) were extracted from knee cartilage of patients underwent total knee arthroplasty, and primary rat chondrocytes were harvested from knee articular cartilage of Sprague Dawley (SD) rats. After the cartilage was digested with 4 mg/mL protease (P5147, Sigma-Aldrich) for 90 min at 37 °C and collagenase D (1.2 mg/mL; Roche) for 60 min and 0.25 mg/mL collagenase P (11213873001, Roche, Mannheim Germany) for 12 h at 37 °C. The primary chondrocytes were seed in culture flasks (75 cm^2^) and cultured with Dulbecco’s modified Eagle’s medium/F-12 (Gibco Life Technologies) containing 10% FBS (Gibco Life Technologies) and 1% penicillin and streptomycin (Gibco Life Technologies) in 5% CO_2_ atmosphere at 37 °C. To ensure the accuracy of the primary chondrocyte phenotype and subsequent experiments, only the second to third passages were used.

### Animals

The study protocol was approved by Institutional Animal Care and Use Committee of Sun Yat-Sen University (SYSU-IACUC-2023-001036). 42 male SD rats (200 ± 10 g, 6 weeks old) purchased from Guangdong Medical Laboratory Animal Center (Guangdong, China) were divided into seven groups (*n* = 6 per group). All SD rats were lived in a specific pathogen-free (SPF) environment and provided with a standard diet.

According to previous studies,^5,73^ the monosodium iodoacetate (MIA, S817623, Macklin, Shanghai, China) models were induced by the injection of 40 µL saline with 1 mg MIA into the right knee joint of 8-week-old SD rats after anesthetization with isoflurane, whereas the left knee joint had a single intra-articular injection of 40 µL saline without MIA as control, which were divided into four groups (*n* = 6) on the basis of MIA treatment time: saline control (Con) and MIA treatment for 1 week, 2 weeks and 3 weeks respectively.

To identify the function of PARP12, we injected XAV-939 (A1877, APEXBIO, Houston, USA) of PARP12 inhibitor and recombinant adenovirus with PARP12 overexpression into the right knee joint of SD rats. On the one hand, XAV-939 was administered in MIA-induced OA rats at doses, frequencies, and durations as described previously.^74^ 50 uL of XAV-939 (0.4 mmol/L) or saline was injected every other day for 2 weeks after MIA injection of 8-week-old SD rats. On the other hand, recombinant adenovirus with PARP12 overexpression was first injected into the knee joint cavity of 7-week-old SD rats three times weekly (30 µL of 10^10^ PFU/mL; Hanbio, Shanghai, China) before the MIA injection for 2 weeks in 8 weeks old. Thus, rats with PARP12 overexpression were divided into two groups (*n* = 6 per group): Ad-NC (OE-NC) and Ad-PARP12-3xflag-EGFP (OE-PARP12). Overall, we randomly divided rats into four groups with six mice per group: Saline, XAV-939, OE-NC and OE-PARP12.

### Micro-CT analysis

The knee joints isolated from rats were fixed in 4% paraformaldehyde. All joints were scanned using the Bruker SkyScan 2211 nano-computed tomography (CT) instrument (Bruker micro-CT, Knotich, Belgium) at a resolution of 8.5 μm. According to the manufacturer’s instructions, we performed Micro-CT using cameras that scanned over 180 degrees of rotation, a voltage of 90 kVp, a current of 450 µA (8-watt output) and a 0.5 mm aluminum filter to prevent beam hardening artifacts. 3D reconstruction images and data analysis were performed using InstaRecon software (Bruker MicroCT, Kontich, Belgium). Condyles of femur, tibial plateau and shaft were defined as the region of interest. BS/TV, BV/TV trabecular thickness and number of condyles of femur and tibial plateau analyses were performed using CTAn software (Version 1.20.8, Bruker MicroCT, Kontich, Belgium).

### RNA extraction and quantitative real-time PCR analysis

Total RNA from chondrocytes was extracted by using RNA-Quick Purification Kit (ES Science, Shanghai, China) following the manufacturer’s instructions. The cDNA was generated by Evo M-MLV RT Premix for qPCR (Accurate Biotechnology, Hunan, China). qPCR was performed by using SYBR^®^ Green Premix Pro Taq HS qPCR kit (Accurate Biotechnology, Hunan, China). RT-qPCR was performed using an ABI ViiA™7 Real-Time PCR System (Applied Biosystems, Foster City, CA, USA) following the manufacturer’s instructions. All reactions were repeated three times and gene expression was calculated using the 2^-ΔΔCt^ method. All primers (Tsingke Biotechnology, Beijing, China) are listed in (Table S2).

### Western blot

Chondrocytes were lysed on ice by RIPA buffer with protease and phosphatase inhibitor cocktail (Beyotime Biotechnology) for 30 min. The protein concentration was detected by the BCA protein assay kit (ThermoFisher). Total protein was further separated by sodium dodecyl sulfate-polyacrylamide gel electrophoresis before was transferred to polyvinylidene fluoride membranes (Millipore, Burlington, MA, USA). Then, the membranes were blocked with protein-free rapid blocking buffer (PS108, Epizyme, Shanghai, China) for 15 min, followed with the incubation of corresponding primary antibodies (Table S3) overnight at 4 °C, and subsequently treated with the corresponding secondary antibodies (1:5 000, Cell Signaling Technology, Danvers, MA, USA) at room temperature for 1 h. Protein bands were detected by an automatic chemiluminescence imaging analysis system (Bio-Rad Laboratories, Hercules, CA, USA) with using ECL solution (Millipore). The intensity of each band was quantified and compared using ImageJ software (NIH, Bethesda, MD, USA). GAPDH is used internal control for total protein.

Mitochondrial proteins are derived by isolating mitochondria from chondrocytes with Cell Mitochondria Kit (C3601, Beyotime) following the manufacturer’s instructions. The Dounce homogenization protocol was used to isolate mitochondria from chondrocytes. Mitochondrial proteins concentrations were measured later and the procedure of western blot were described above. COXIV is used internal control for Mitochondrial proteins.

For HA-ubiquitylated proteins, chondrocytes were transfected with HA-ubiquitin using PolyFect transfection reagent (301105, Qiagen, Hilden, Germany). After transfected 24 h, isolating mitochondria and western blot analyses to detect HA-ubiquitin conjugated proteins using an anti-HA antibody (51064-2-AP, Proteintech, Wuhan, China) were performed as described above.

### Immunofluorescence

Chondrocytes were washed with PBS and fixed with 4% paraformaldehyde for 30 min, permeabilized with 0.1% Triton X-100 (Sigma) for 10 min and then blocked with 1% bovine serum albumin (Sigma) for 1 h at room temperature. Chondrocytes were then incubated with primary antibodies (Table S3) at 4 °C overnight. Chondrocytes were washed and further incubated with secondary antibodies conjugated to fluorescent Cy5 dye (1:100; Abcam) at room temperature for 1 h. The nuclei were stained with 4,6-diamidino-2-phenylindole dihydrochloride (DAPI) for 5 min at room temperature. Immunofluorescence images were visualized by a confocal microscope (LSM 780, Zeiss, Oberkochen, Germany).

### Colocalization of mitochondria and lysosomes

Chondrocytes were stained with MitoTracker Red (C1035, Beyotime) and LysoTracker Green (C1047S, Beyotime) following manufacturer’s instructions, and then Images were collected using the FV3000 confocal laser scanning biological microscope in two channels (Olympus, Tokyo, Japan).

### Keima with mitochondrial localization signal (Mito-Keima)

Mito-Keima is a mitochondrially localized pH-indicator protein.^75^ The method used to detect lysosomal delivery of Mito-Keima has been described.^76^ In brief, we calculated the area of dots with a high red/green ratio, indicating localization of Mito-Keima in lysosomes.

### Histological analysis, scoring system and immunohistochemistry

The damaged and undamaged cartilage of patients and the rat joints were fixed for 3 d in 4% paraformaldehyde (Sigma-Aldrich) at room temperature and decalcified in 15% EDTA (pH 7.4) for 21 d at 37 °C. Samples were embedded in paraffin after dehydration with graded ethanol series and vitrification with xylene. Cartilage of patients were cut into 5 μm sections, and then stained with Alcian Blue, Safranin O, or Toluidine Blue. All rat joints were cut into 5 μm sections, and then stained with 0.1% Safranin O solution and 0.001% Fast Green solution (Sigma-Aldrich). OARSI grade was evaluated the degree of rat cartilage degeneration by two researchers in a blinded manner.^5,77^

For immunohistochemistry, COL2A1 (1:200, 28459-1-AP, Proteintech), MMP13 (1:200, 18165-1-AP, Proteintech) and PARP12 (1:100, C28501, Signalway Antibody) according to the manufacturer’s instruction were conducted after deparaffinization with xylene and hydration with graded ethanol series. All positively stained cells were counted in the Condyles of femur and tibial plateau region, and the proportion of positive cells was evaluated using ImageJ software (NIH, Bethesda, MD, USA).

### RNA interference and overexpression

Primary human chondrocytes (PHCs) were transfected using Lipofectamine 3000 (Invitrogen, Carlsbad, USA) according to the manufacturer’s instructions. Chondrocytes were transfected with PARP12 KD shRNAs or overexpression (OE), ISG15 KD shRNAs or OE, IRF1 KD shRNAs or OE, KD negative control (NC) shRNA or OE NC (Tsingke Biotechnology, Beijing, China). The specific sequences are listed in (Table S4).

### Immunoprecipitation (IP) and Proteomics of mass spectrometry (MS)

In order to find the interacting proteins with PARP12, according to the manufacturer’s instructions of Biolinkedin^®^ classic Protein A/G IP kit (IK-1004, Biolinkedin), total protein was incubated with 1 μg of normal rabbit IgG (GB111738, Servicebio, Wuhan, China) as control or 1 μg of anti-PARP12 antibody (ab241967, Abcam) overnight at 4 °C after the extraction of PHCs using combination of Lysis buffer, Phenylmethanesulfonyl fluoride, and phosphatase inhibitors at a ratio of 100:2:1. The mixtures of protein and antibody were subsequently incubated with 25 μL of pre-washed Protein A/G beads in gentle rotation at room temperature for 2 h. Then, the immunoprecipitates were separated by SDS-PAGE on 10% agarose gel and stained by silver staining (P0017S, Beyotime). Mass spectrometry (Novogene) and western blot were performed.

To verify the interaction between PARP12 with ISG15, MFN1 or MFN2, and analyze ISGylation, SUMOylation and ubiquitylation of MFN1/2, the related antibodies of IP and western blot are shown in (Table S3).

To test if SUMO2/3-dependent ubiquitin conjugation to cellular proteins is affected in chondrocytes, we immunoprecipitated SUMO2/3-conjugated proteins using SUMO2/3 affinity beads from chondrocytes with KD-ISG15 or KD-NC, and detected levels of SUMO2/3 and ubiquitin conjugates using anti-SUMO2/3 and anti-ubiquitin antibodies in the same samples. According to the manufacturer’s instructions of SUMOylation 2/3 affinity beads (BK162, Cytoskeleton, Inc.), SUMO2/3-conjugated proteins in chondrocytes lysates were immunoprecipitated. Then, western blot of SUMO2/3 conjugated proteins was performed.

### Transcription factor prediction and chromatin immunoprecipitation assay

The NCBI gene database was used to search for the promoter region of PARP12 and the JASPAR 2020 TFBS track of the University of California, Santa Cruz (UCSC) Genome Browser for TFs. According to the predicted score of TFs, previous associations with OA and was upregulated significantly in damaged cartilage samples of our mRNA-seq analyses (10.07-fold), we focused on IRF1 and predicted its binding sites in the promoter region of PARP12 using the JASPAR database. The GEPIA database also indicated that the expression of PARP12 was positively correlated with IRF1. Thus, we further identified whether IRF1 directly targets the promoter region of PARP12.

Cellular lysates were harvested from PHCs, and ChIP assay was conducted using an IP Kit of SimpleChIP Enzymatic Chromatin (9002S, CST) according to the manufacturer’s instructions. Briefly, PHCs were fixed in 1% formaldehyde at room temperature for 10 min to cross link DNA and protein. Then, they were treated with Buffer A, Buffer B, Micrococcal Nuclease (10011, CST), and ChIP buffer in this sequence to extract fragmented chromatins. They were incubated overnight with normal rabbit IgG (2729, CST) as control or anti-IRF1 antibody (11335-1-AP, Proteintech) at 4 °C. The mixtures of protein-DNA and antibody were then incubated with Protein G Agarose Beads of ChIP-Grade (9007, CST) at 4 °C for 3 h with shaking. After washing three times with low and once with high-salt, the protein-DNA crosslinks of the immunoprecipitates were uncoupled by reaction with proteinase K for 2 h, and DNA was purified with DNA purification columns. Purified DNA was subjected to qRT-PCR with the primers (Tsingke Biotechnology, Beijing, China) were listed in (Table S2).

### Transmission electron microscopy (TEM)

Chondrocytes were fixed with 2.5% glutaraldehyde (pH 7.4, Sigma-Aldrich) for 2 h at room temperature. After centrifugation at low speed (1 200 r/min, 3 min), a mung-bean-sized cell mass was observed at the bottom of the tube. Cells were treated and fixed overnight in 2.5% glutaraldehyde and after 2 h harvested in 1% osmium tetroxide (Structure Probe, Inc, USA) at 4 °C. Following dehydration, infiltration, embedding, and sectioning, images were captured using a Tecnai G2 Spirit Twin TEM (FEI Company, USA).

### Reactive Oxygen Species (ROS) staining

Chondrocytes were stained with diluted 2′,7′-dichlorofluorescin diacetate (DCFH-DA) and incubated at 37 °C for 20 min according to the manufacturer’s protocol (S0033M, Beyotime). Then, the chondrocytes were washed twice with phosphate-buffered saline (PBS, Servicebio, Wuhan, China) to remove the excess DCFH-DA. Samples were detected by using an inverted fluorescence microscope (Olympus), and the intensity was quantitated by the ImageJ software.

### Flow cytometry

Apoptosis was evaluated by staining PHCs with both Annexin V-FITC and PI following the manufacturer’s protocol (FXP022-100, 4 A Biotech, Suzhou, China). PHCs that were positively stained with Annexin V-FITC and negatively stained for PI were considered apoptosis. PHCs that were positively stained for both Annexin V-FITC and PI were considered necrosis. The PHCs were stained with 5 µL Annexin V-FITC and 10 µL PI and then analysed. Analyses were performed using FlowJo Flow Cytometry Analysis Software ((Tree Star).

### JC-1 staining

JC-1 staining was performed using an enhanced mitochondrial membrane potential assay kit with JC-1 (C2003S, Beyotime) according to the manufacturer’s instructions. Chondrocytes were in six-well plates, then washed twice with PBS, mixed thoroughly with JC-1 staining working solution and incubated for 20 min at 37 °C. Chondrocytes were then washed twice with JC-1 staining buffer. Subsequently, samples were observed under an inverted fluorescence microscope (Olympus). Red represents normal mitochondria, whereas green suggests damaged mitochondria. The ratio of red to green fluorescence were calculated to evaluate mitochondrial membrane potential.

### ATP detection

ATP levels were detected by using a firefly luciferase-based ATP detection kit (S0026, Beyotime). After being washed twice with PBS, chondrocytes were lysed using ATP detection lysis buffer, followed by centrifugation at 12 000 × *g* for 5 min at 4 °C, and the supernatant of lysate was collected. Protein of the supernatant concentration was determined for each sample and corrected for uniformity. Then, 20 μL of the supernatant or standard sample was seed in a 96-well plate that was opaque to light and mixed with 100 μL of ATP detection solution. Luminescence was immediately measured in relative light units (RLU) (nmol/mg) using a Turner Biosystems luminometer (Tecan, Switzerland). Finally, the ATP level of each sample was determined according to the RLU value of the standard sample.

### Cell activity assay

Cell Counting Kit-8 (CCK-8) (C0038, Beyotime) was used to detect chondrocyte activity, PHCs were counted and added into 96-well plates sequentially with 100 μL per well, the cells were treated with appropriate reagents, with a concentration of 10% of the CCK-8 working solution incubating for 1–4 h then the absorbance at 450 nm was measured using an enzyme marker.

### Mito-tracker staining

Mitochondria staining was performed using Mito-Tracker Red CMXRos (C1035, Beyotime). Briefly, chondrocytes were incubated with Mito-Tracker Red (100 nmol/L) for 30 min at 37 °C. Then DAPI Staining Solution (C1006, Beyotime) was used to detect nucleus according to the manufacturer’s protocol. Chondrocytes washed twice with PBS. Subsequently, samples were observed under an inverted fluorescence microscope (Olympus). The intensity of mitochondria staining was quantitated by the ImageJ software.

### Molecular docking

The crystal structures of PARP12 and ISG15 were obtained from the Protein Data Bank (https://rcsb.org). The PARP12 and ISG15 docking was predicted by the ClusPro Server, according to a previous study.^78^

### Statistical analysis

All experiments were performed in at least three biological replicates. Experimental data are shown as the mean ± SD. Statistical significances were calculated with Student’s t-test for comparisons between 2 groups and ANOVA for multiple group comparisons as showed in figure legends. The relationship between IRF1 and PARP12 was analyzed using simple linear regression. The relationships between PARP12 or IRF1 expression and baseline characteristics of patients with OA were evaluated using the chi-squared test and Spearman’s correlation analysis. R^2^ < 0.16 means a low linear correlation, 0.16 ≤ R^2^ < 0.49 means a significant correlation, 0.49 ≤ R^2^ < 1 means a high linear correlation. *P* values were considered significant at *P < 0.05, **P < 0.01, ***P < 0.001. Data were analyzed using GraphPad Prism 8.0 (GraphPad, La Jolla, CA, USA) or SPSS v26.0 (SPSS, Inc., Chicago, IL, USA).

## Supplementary information

IRF1-mediated upregulation of PARP12 promotes cartilage degradation by inhibiting PINK1/Parkin dependent mitophagy through ISG15 attenuating ubiquitylation and SUMOylation of MFN1/2

## Data Availability

All data needed to evaluate the conclusions in the paper are present in the paper and/or the Supplementary Materials. The data that support the findings of this study are available from the corresponding author upon reasonable request.
