# Supplementary material for: IRF1-mediated upregulation of PARP12 promotes cartilage degradation by inhibiting PINK1/Parkin dependent mitophagy through ISG15 attenuating ubiquitylation and SUMOylation of MFN1/2
Source: Bone Res. 2024 Oct 28;12:63. doi: 10.1038/s41413-024-00363-3 (PMC11514270; doi:10.1038/s41413-024-00363-3)
Supplement: Supplementary file 1 — IRF1-mediated upregulation of PARP12 promotes cartilage degradation by inhibiting PINK1/Parkin dependent mitophagy through ISG15 attenuating ubiquitylation and SUMOylation of MFN1/2 [file 41413_2024_363_MOESM1_ESM.docx]

**Supplementary Material**

**Supplementary Tables**

**Table S1 Spearman correlation analysis between PARP12 expression and the baseline characteristics patients with osteoarthritis (n = 30)**

| Variables | PARP12 expression | |
| --- | --- | --- |
|  | Spearman | *P* value |
| Age | 0.477 | 0.008 |
| Sex | -0.089 | 0.606 |
| Weight | 0.235 | 0.211 |
| Height | -0.214 | 0.256 |
| Body Mass Index | 0.347 | 0.06 |
| Obesity gradation | 0.303 | 0.103 |
| Affected side | 0.102 | 0.591 |
| Disease duration | 0.403 | 0.027 |
| Kellgren–Lawrence gradation | 0.723 | <0.001 |

Note: *P* < 0.05 considered significant.

**Table S2** Primers and sequences used in this study.

| **Primers for qPCR** | | |
| --- | --- | --- |
| Human PARP8 | F | GGGATGTGTTCAAGGCAAGAG |
|  | R | CCGCCAACGTAGGTAAAAGTAA |
| Human PARP12 | F | GCCATGACTTACGGTGCTACC |
|  | R | CCAAACTCATCACTCCAGTACCA |
| Human PARP14 | F | TGTTAGTGGAGAACATAAGTGGC |
|  | R | TGAATGGTGCTTGGTACAATCAT |
| Human PARP16 | F | CCTCAAAGGTCCTGACAATCC |
|  | R | CTAGGCGGCTACCATGAAATG |
| Human COL2A1 | F | CCAGATGACCTTCCTACGCC |
|  | R | TTCAGGGCAGTGTACGTGAAC |
| Human Aggrecan | F | GATGTTCCCTGCAATTACCACCTC |
|  | R | TGATCTCATACCGGTCCTTCTTCTG |
| Human SOX9 | F | GGAGATGAAATCTGTTCTGGGAATG |
|  | R | TTGAAGGTTAACTGCTGGTGTTCTG |
| Human MMP13 | F | TCCTGATGTGGGTGAATACAATG |
|  | R | GCCATCGTGAAGTCTGGTAAAAT |
| Human RUNX2 | F | CACTGGCGCTGCAACAAGA |
|  | R | CATTCCGGAGCTCAGCAGAATAA |
| Human ADAMTS4 | F | GAGGAGGAGATCGTGTTTCCA |
|  | R | CCAGCTCTAGTAGCAGCGTC |
| Human Bcl2 | F | GGTGGGGTCATGTGTGTGG |
|  | R | CGGTTCAGGTACTCAGTCATCC |
| Human Bax | F | CCCGAGAGGTCTTTTTCCGAG |
|  | R | CCAGCCCATGATGGTTCTGAT |
| Human LC3B | F | TTATTCGAGAGCAGCATCCAACC |
|  | R | CCGTTCACCAACAGGAAGAAGG |
| Human p62 | F | GACTACGACTTGTGTAGCGTC |
|  | R | AGTGTCCGTGTTTCACCTTCC |
| Human Beclin1 | F | ACCTCAGCCGAAGACTGAAG |
|  | R | AACAGCGTTTGTAGTTCTGACA |
| Human GAPDH | F | GGAGCGAGATCCCTCCAAAAT |
|  | R | GGCTGTTGTCATACTTCTCATGG |
| Primers for predicted IRF1 binding sites on PARP12 promoter | | |
| Negative Control PCR1 | F | GATGCACCGAGGCGAAAG |
|  | R | GGACAACTGACGGGAGC |
| PARP12 PCR2 | F | AGCTGCGGACCGGGG |
|  | R | CTCGCAGGGTGGAGACGC |
| PARP12 PCR3 | F | ACTGTTCCCACGTAACCCTAC |
|  | R | CCCAGCAATCTTTCCTCTCGGA |
| PARP12 PCR4 | F | ACCGTATACCGTAAGTACTCTCCC |
|  | R | AAGGGTAGTTGAGAGGAGGCA |

**Table S3** Primary antibodies used in this study.

| **Primary antibodies** | **Article number and manufacturer** | **Application** |
| --- | --- | --- |
| PARP12 | ab241967, Abcam | Western blots, IF and IP in human |
| PARP12 | C28501, Signalway Antibody | Western blots, IHC and IF in rat |
| COL2A1 | ab188570, Abcam | Western blots in human and rat |
| COL2A1 | 28459-1-AP, Proteintech | IF in human, IHC in human and rat |
| MMP13 | 18165-1-AP, Proteintech | Western blots in human and rat, IF in human, IHC in human and rat |
| p62 | GB11531, Servicebio | Western blots in human and rat, IF in human and rat, IHC in human |
| LC3B | 43566, Cell Signaling Technology | Western blots in human and rat, IF and IHC in human |
| PARP16 | bs-9623R, Bioss | Western blots in human |
| Aggrecan | 13880-1-AP, Proteintech | Western blots in human and rat |
| RUNX2 | GB13264, Servicebio | Western blots in human and rat |
| Bcl2 | 68103-1-Ig, Proteintech | Western blots in human and rat |
| Bax | 50599-2-Ig, Proteintech | Western blots in human and rat |
| MFN1 | 13798-1-AP, Proteintech | Western blots and IP in human, Western blots in rat |
| MFN2 | 12186-1-AP, Proteintech | Western blots and IP in human, Western blots in rat |
| MFN1/2 | ab57602, Abcam | Western blots and IP in human, IF in human |
| ISG15 | 15981-1-AP, Proteintech | Western blots, IP and IF in human |
| NLRP3 | GB114320, Servicebio | Western blots in human and rat |
| ASC | 10500-1-AP, Proteintech | Western blots in human |
| ASC | ab307560, Abcam | Western blots in rat |
| Caspase1 | 22915-1-AP, Proteintech | Western blots in human and rat |
| IL-1β | GB11113, Servicebio | Western blots in human and rat |
| ubiquitin | 10201-2-AP, Proteintech | Western blots in human |
| SUMO2/3 | 11251-1-AP, Proteintech | Western blots and IF in human |
| HA | 51064-2-AP, Proteintech | Western blots in human |
| PINK1 | 23274-1-AP, Proteintech | Western blots in human and rat |
| Parkin | 14060-1-AP, Proteintech | Western blots in human and rat |
| IRF1 | 11335-1-AP, Proteintech | Western blots and IP in human |
| Wnt3a | 26744-1-AP, Proteintech | Western blots in human |
| Axin2 | 20540-1-AP, Proteintech | Western blots in human |
| COXIV | 11242-1-AP, Proteintech | Western blots in human |
| GAPDH | HRP-60004, Proteintech | Western blots in human and rat |

**Table S4** knockdown sequence of PARP12, ISG15 and IRF1 in human.

| PARP12 | KD-01 | CAGAGTTTCTAGATACAATTT |
| --- | --- | --- |
|  | KD-02 | GAAAGCACAACTACGAGTTAG |
|  | KD-03 | GCTCCATATCTGCCAGTATT |
|  | KD-04 | CGAAGAGCATCCCAGACTATT |
| ISG15 | KD-01 | CTGAGCATCCTGGTGAGGAAT |
|  | KD-02 | CATGTCGGTGTCAGAGCTGAA |
|  | KD-03 | CCACCTGAAGCAGCAAGTGAG |
|  | KD-04 | GACCTGTTCTGGCTGACCTTC |
| IRF1 | KD-01 | GGCTAGAGATGCAGATTAATT |
|  | KD-02 | TGGCTAGAGATGCAGATTAAT |
|  | KD-03 | CCTCTGTCTATGGAGACTTTA |
|  | KD-04 | GCGTGTCTTCACAGATCTGAA |
| Axin2 | KD-01 | GCGTGGATACCTTAGACTTCT |
|  | KD-02 | GGACTTGTGCCGACTTCAAGT |
|  | KD-03 | GCAGCGAGTATTACTGCTACT |
|  | KD-04 | CCGATGTATGAAGGCCGGATT |

**Supplementary Figures**


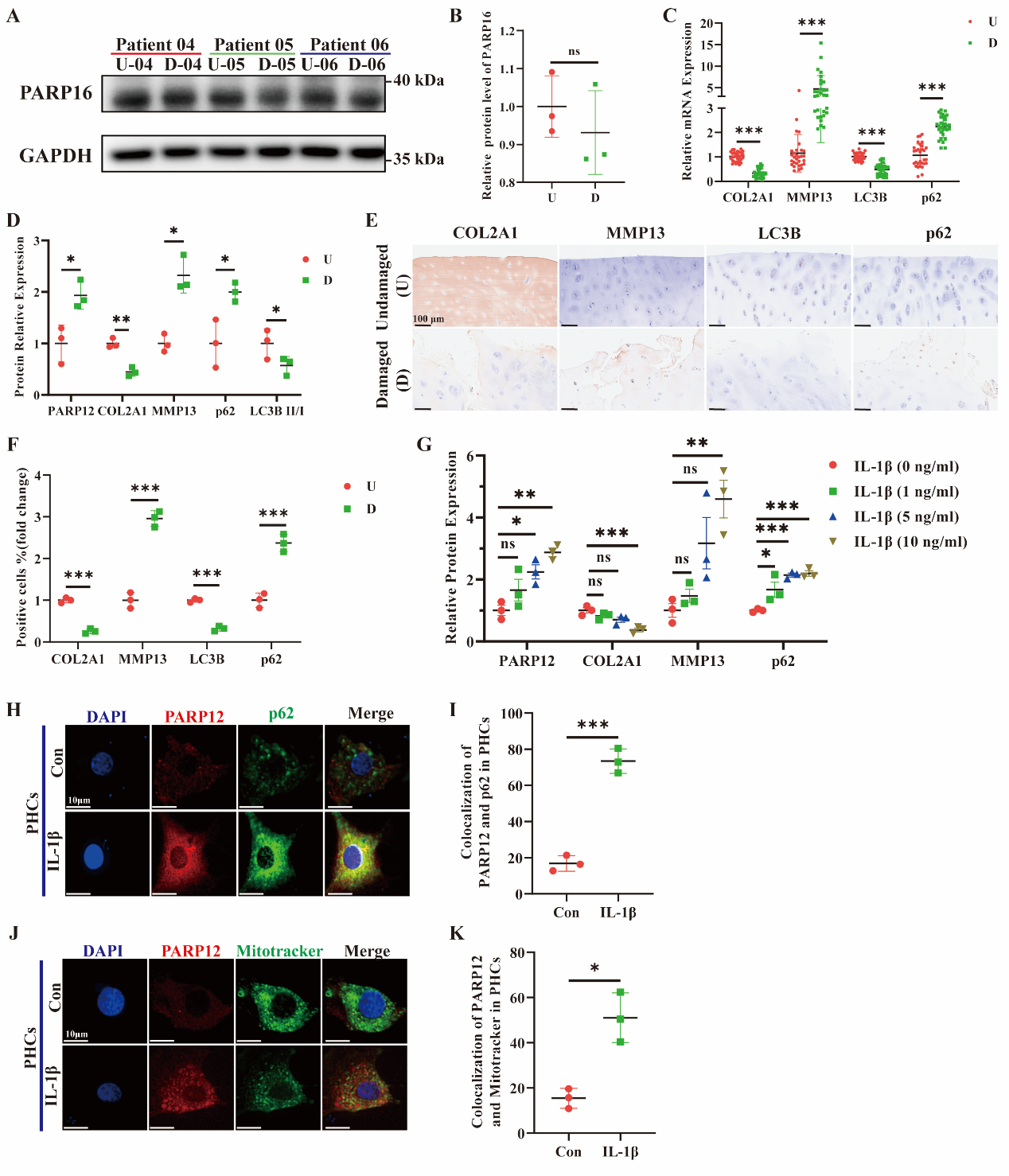


**Figure S1. The damaged areas of knee OA cartilage show significant degeneration and a decline in autophagy capabilities, and PARP12 being associated with mitophagy.** (A, B) Western blot and quantification analysis of PARP16 between U and D cartilage tissues. (C) Quantitative PCR analysis of COL2A1, MMP13, LC3B and p62 in U and D cartilage tissues. n = 30 per group. (D) Western blot quantification of PARP12, COL2A1, MMP13, p62, and LC3B II/I between U and D cartilage tissues via ImageJ. n=3 per group. (E) IHC staining of COL2A1, MMP13, LC3B and p62 between U and D cartilage tissues. Scale bars: 100 µm. (F) Quantification of COL2A1, MMP13, LC3B and p62 positive chondrocytes based on staining results in (I). n = 3 per group. (G) Western blot quantification of PARP12, COL2A1, MMP13, p62, and LC3B II/I in primary chondrocytes (PHCs) treated with different concentrations of IL-1β (0, 1, 5, or 10 ng/mL) for 24 h via ImageJ. n=3 per group. (H-K) Colocalization and quantification analysis of PARP12 and p62 or mitotracker in PHCs. Scale bars: 10 µm. n=3 per group. Data were presented as mean ± SD. Paired t-test was used for statistical analysis of (B, C, D, F, I, K). One-way analysis of variance (ANOVA) with Dunnett’s multiple comparisons test was used for statistical analysis of (G). **P* < 0.05, ***P* < 0.01, and ****P* < 0.001.


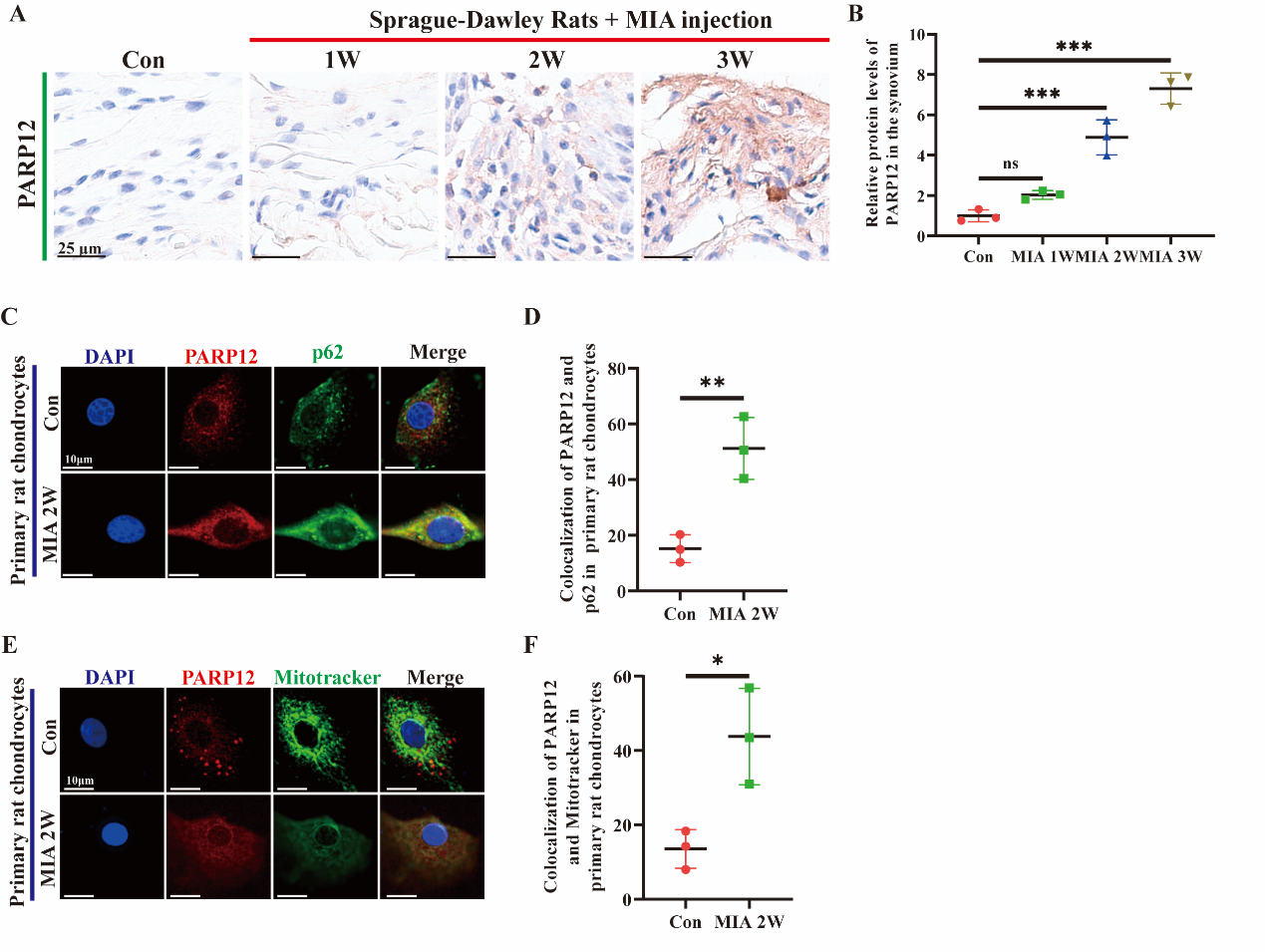


**Figure S2. The expression pattern of PARP12 in the MIA-induced OA model in rats.** (A, B) IHC staining and quantification analysis of PARP12 in synovium of Sprague–Dawley rats. Scale bars: 25 µm. n=3 per group. (C-F) Colocalization and quantification analysis of PARP12 and p62 or mitotracker in chondrocytes of Sprague–Dawley rats injected with saline control (con) or MIA for 2 weeks. Scale bars: 10 µm. n=3 per group. Data were presented as mean ± SD. One-way analysis of variance (ANOVA) with Dunnett’s multiple comparisons test was used for statistical analysis of (B). Paired t-test was used for statistical analysis of (D, F). **P* < 0.05, ***P* < 0.01, and ****P* < 0.001.


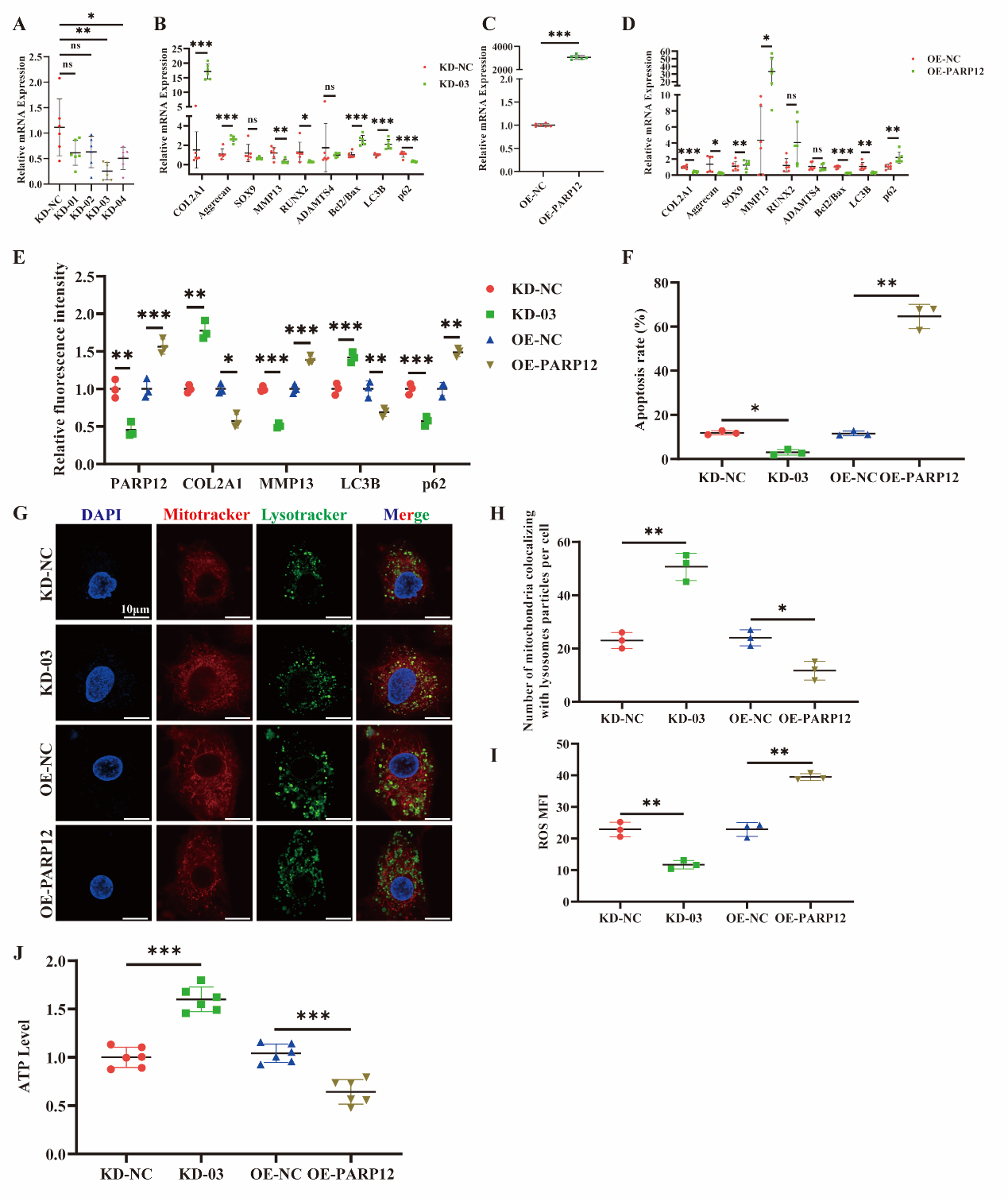


**Figure S3. Targeting the expression of PARP12 affects mitophagy and OA-related degeneration in primary human chondrocytes (PHCs).** (A) Expression of PARP12 in PHCs transfected with *PARP12* knockdown (KD) shRNAs or negative control (NC) shRNA. n = 6 per group. (B). Quantitative PCR analysis of COL2A1, aggrecan, SOX9, MMP13, RUNX2, ADAMTS4, Bcl2/Bax, LC3B and p62 in PHCs infected with *PARP12* KD-03 shRNA or KD-NC shRNA. n = 6 per group. (C) Expression of PARP12 in PHCs transfected with PARP12-OE or OE-NC adenoviruses. n = 6 per group. (D) Quantitative PCR analysis of COL2A1, aggrecan, SOX9, MMP13, RUNX2, ADAMTS4, Bcl2/Bax, LC3B and p62 in PHCs infected with PARP12-OE or OE-NC adenoviruses. n = 6 per group. (E) Immunofluorescence quantification of PARP12, COL2A1, MMP13, LC3B and p62 in PHCs with PARP12 knockdown or overexpression via ImageJ. n=3 per group. (F) Quantification analysis of chondrocytes apoptosis. n = 3 per group. (G) MitoTracker Red and LysoTracker Green staining of PHCs was observed by confocal microscopy. Scale bars: 10 µm. (H) The colocalization of mitochondria with lysosomes was quantified. n = 3 per group. (I) Quantification of ROS using ImageJ. n = 3 per group. (J) Quantification of the ATP level. n = 6 per group. Data are presented as the mean ± SD. One-way analysis of variance with Dunnett’s multiple comparisons test (A) and paired *t*-test (B-F, H, J) were used for statistical analysis. **P* < 0.05, ***P* < 0.01, and ****P* < 0.001.

**
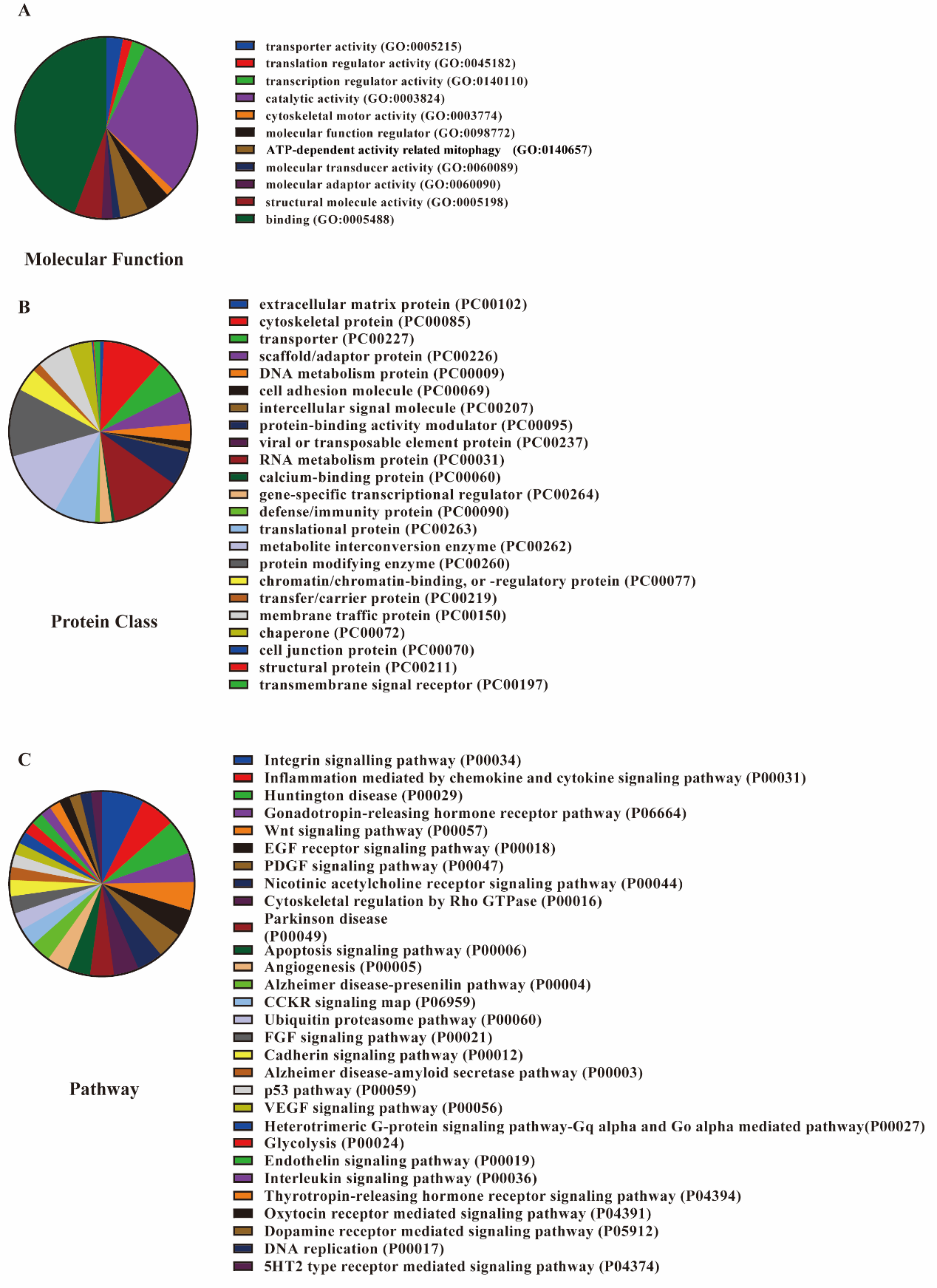
**

**Figure S4. Bioinformatics analysis of proteins of interacting with PARP12.** (A) Molecular function of proteins of interacting with PARP12. (B) Protein class of interacting with PARP12. (C) Pathway of proteins of interacting with PARP12.


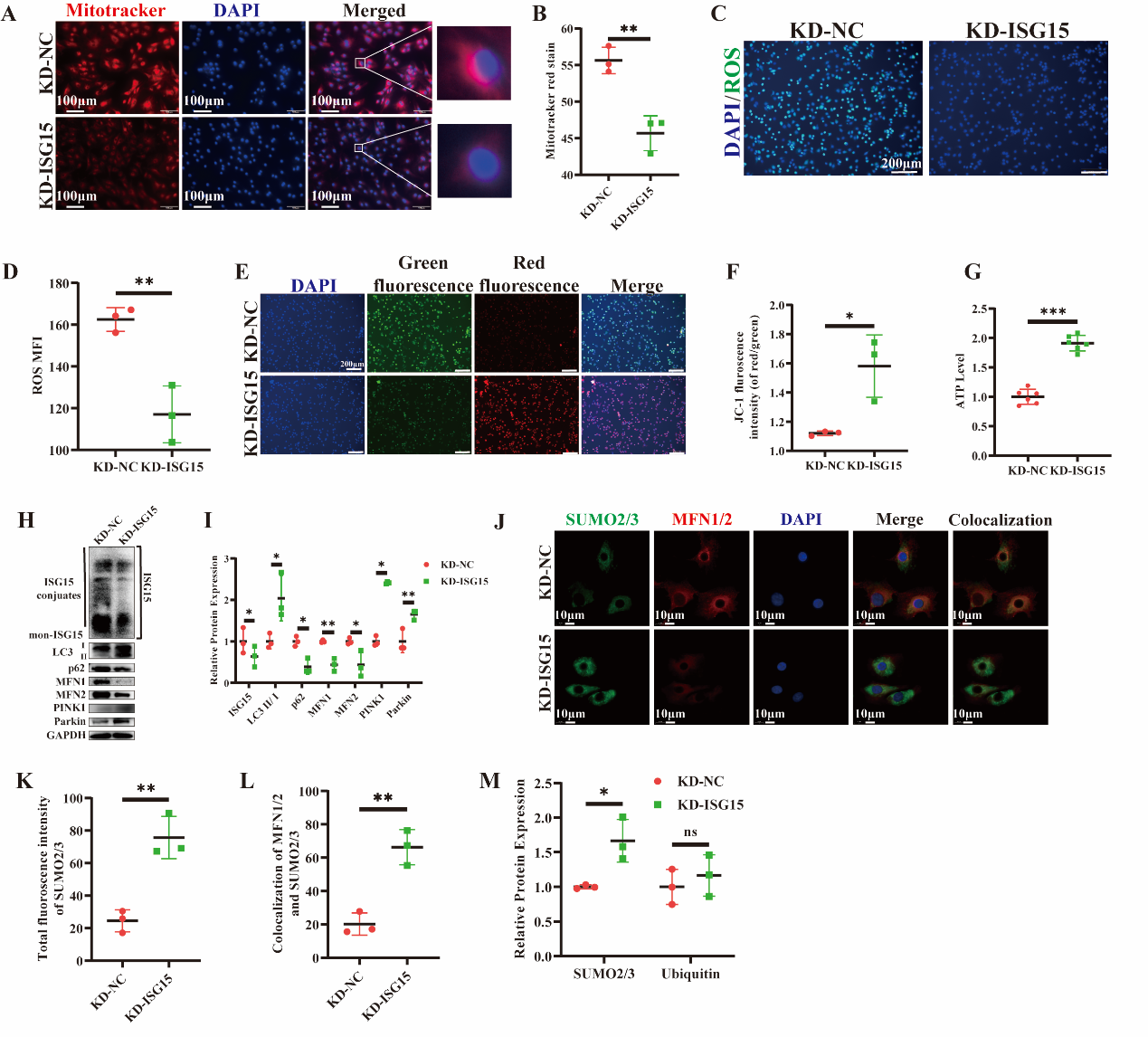


**Figure S5. ISG15 interfering with ubiquitylation and SUMOylation of MFN1/2 and then suppressed PINK1/Parkin-dependent mitophagy.** (A) Representative images of mitotracker stain in PHCs following transfection with ISG15 knockdown (KD) shRNA or negative control (NC) shRNA adenoviruses. The white box indicates the amplified region showing a single cell exhibiting the typical staining pattern of mitochondria. Scale bars: 100 µm. (B) Quantification of mitotracker red stain of (A) using ImageJ. n = 3 per group. (C) Reactive oxygen species (ROS) staining in PHCs following transfection with KD-ISG15 or KD-NC. Scale bars: 200 µm. (D) Quantification of ROS of (C) using ImageJ. n = 3 per group. (E) JC-1 staining in PHCs following transfection with KD-ISG15 or KD-NC. Scale bars: 200 µm. (F) Quantification of JC-1 staining of (E) using ImageJ. n = 3 per group. (G) Quantification of ATP level in PHCs following transfection with KD-ISG15 or KD-NC. n = 6 per group. (H) Western blot analysis of ISG15, LC3B, p62, MFN1, MFN2, PINK1 and Parkin in PHCs following transfection with KD-ISG15 or KD-NC. (I) Protein quantification of (H) using ImageJ. n = 3 per group. (J) Immunofluorescence of SUMO2/3 and MFN1/2 and their colocalization in PHCs following transfection with KD-ISG15 or KD-NC. Scale bars: 10 µm. (K, L) Quantification of fluorescence intensity of SUMO2/3 and colocalization of SUMO2/3 and MFN1/2 of (J) using ImageJ. n = 3 per group. (M) Quantification of SUMO2/3 and ubiquitin via ImageJ. n = 3 per group.


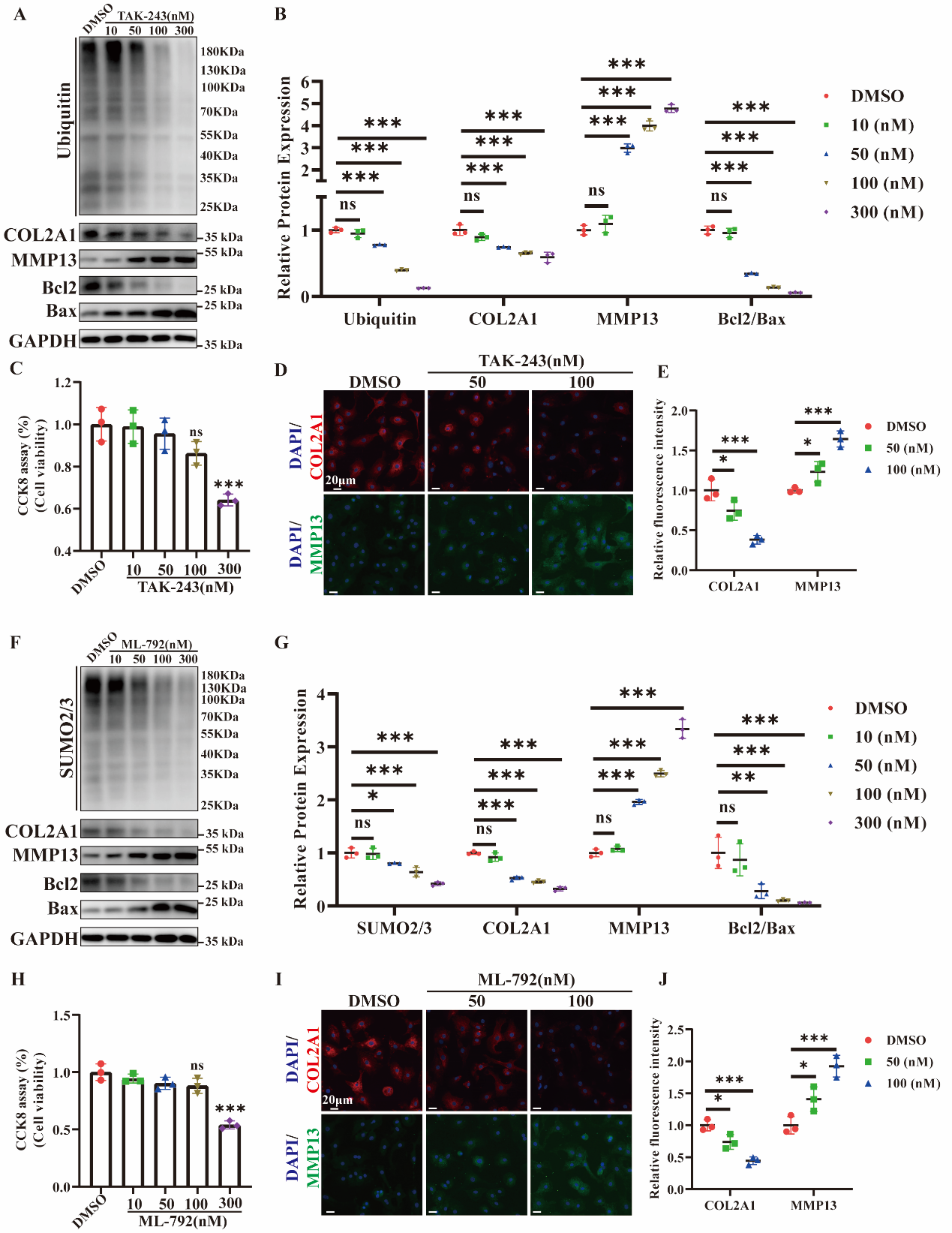


**Figure S6. Evaluating the function of ubiquitination and SUMOylation in OA.** (A, B) Western blot and quantification analysis of ubiquitin, COL2A1, MMP13, Bcl2 and Bax in PHCs treated with different concentrations of ubiquitination inhibitor TAK-243. n = 3 per group. (C, H) Cell viability was analyzed using the CKK-8 assay. n = 3 per group. (D, E) Immunofluorescence and protein quantification of COL2A1 and MMP13 in PHCs following TAK-243 treatment. n = 3 per group. Scale bars: 20 µm. (F, G) Western blot and quantification analysis of ubiquitin, COL2A1, MMP13, Bcl2 and Bax in PHCs treated with different concentrations of ubiquitination inhibitor TAK-243. n = 3 per group. (I, J) Immunofluorescence and protein quantification of COL2A1 and MMP13 in PHCs following ML-792 treatment. n = 3 per group. Scale bars: 20 µm.


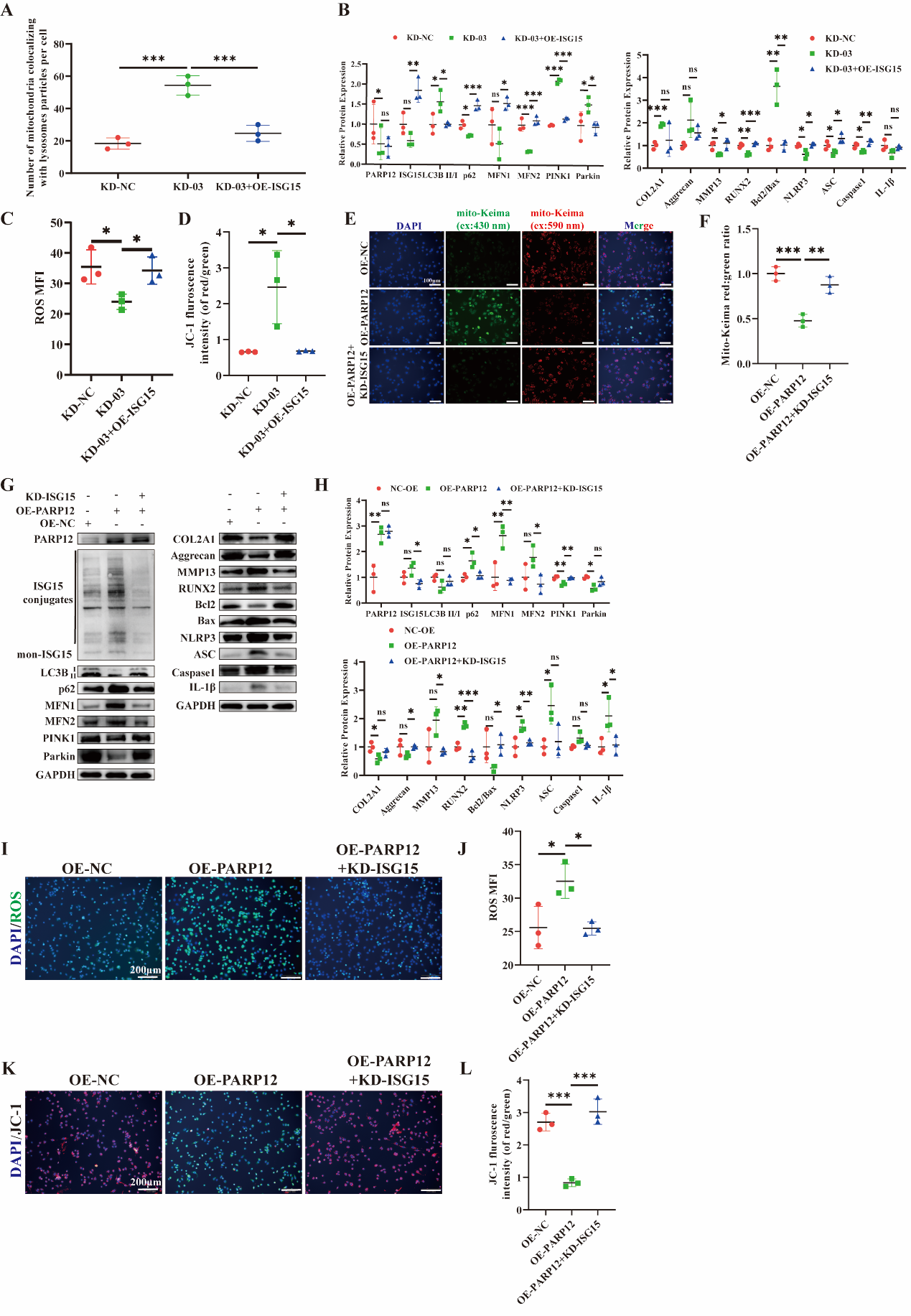


**Figure S7. The rescue experiment of PARP12 and ISG15.** (A) The colocalization of mitochondria with lysosomes was quantified. n = 3 per group. (B) Protein quantification of PARP12, ISG15, LC3B, p62, MFN1, MFN2, PINK1, Parkin, COL2A1, Aggrecan, MMP13, RUNX2, Bcl2, Bax and NLRP3 inflammasome activity with PARP12 knockdown and ISG15 overexpression. n=3 per group. (C) ROS quantification via ImageJ. n=3 per group. (D) JC-1 staining quantification via ImageJ. n=3 per group. (E) The represent images of PHCs transfected with mito-Keima. Scale bars: 100 µm. (F) The relative ratio of red to green fluorescence area per cell of (E) was quantified. n=3 per group. (G) Western blot analysis of PARP12, ISG15, LC3B, p62, MFN1, MFN2, PINK1, Parkin, COL2A1, Aggrecan, MMP13, RUNX2, Bcl2, Bax and NLRP3 inflammasome activity in PHCs with PARP12 overexpression and ISG15 knockdown. n=3 per group. (H) Protein quantification of (G) via ImageJ. n=3 per group. (I) ROS staining with PARP12 overexpression and ISG15 knockdown. Scale bars: 200 µm. (J) ROS quantification of (I) via ImageJ. n=3 per group. (K) JC-1 staining in PHCs with PARP12 overexpression and ISG15 knockdown. Scale bars: 200 µm. (L) JC-1 staining quantification of (K) via ImageJ. n=3 per group. Data were presented as mean ± SD. One-way analysis of variance followed by Tukey’s multiple comparison test (A-D, F, H, J, L) was used for statistical analysis. **P*<0.05, ***P*<0.01, ****P*<0.001.


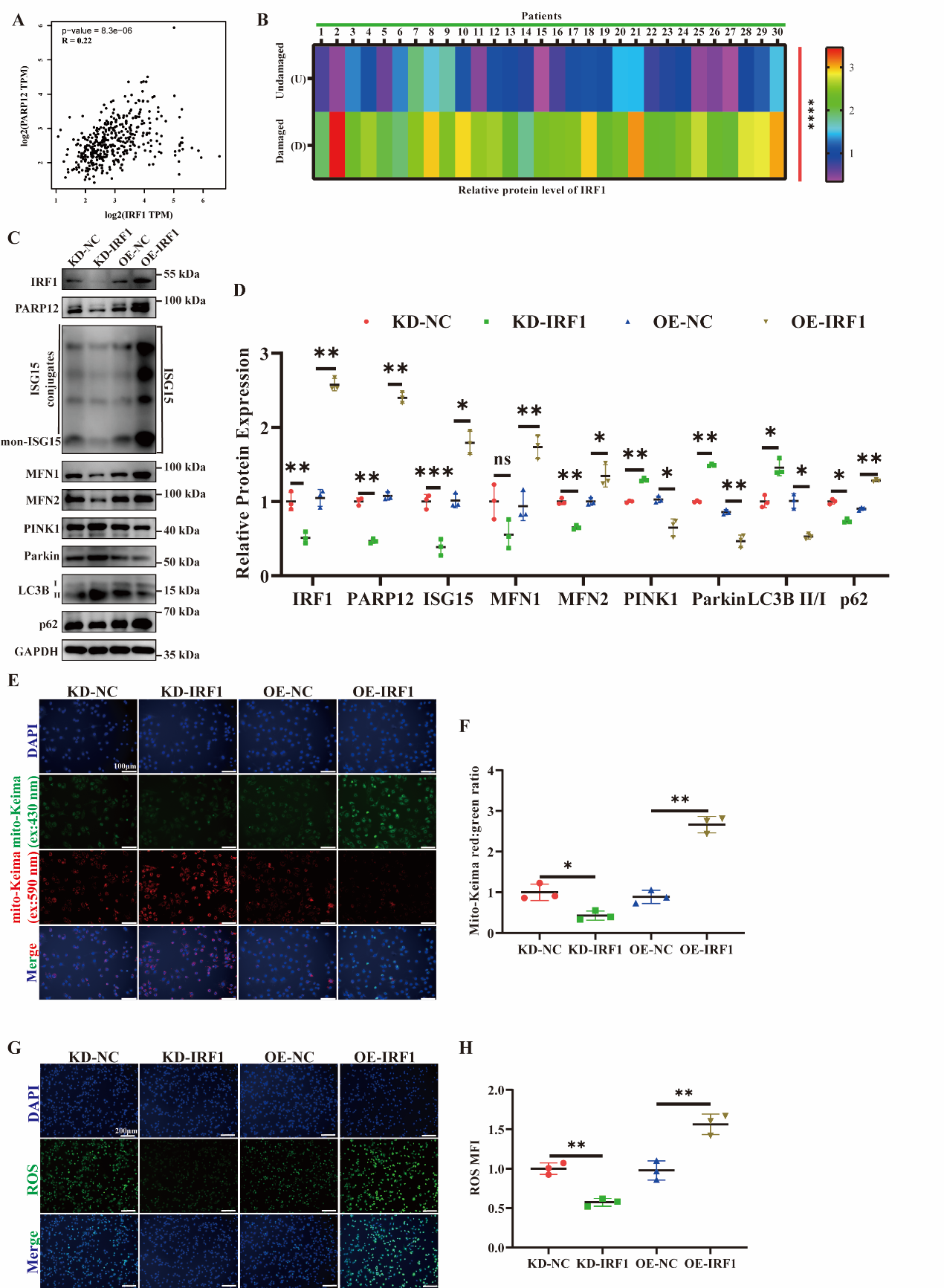


**Figure S8. IRF1 promoting the expression of PARP12, ISG15, MFN1/2 and suppressing mitophagy.** (A) The expression of PARP12 is positively correlated with IRF1 in GEPIA database. (B) Heat map of relative protein level of IRF1 in the undamaged (U) and damaged (D) cartilage tissues of different patients with OA. n=30 per group. (C, D) Western blot and quantification of IRF1, PARP12, ISG15, MFN1/2, PINK1/Parkin, LC3B-II/I and p62 in PHCs following either knockdown or overexpression of IRF1. n = 3 per group. (E) PHCs were transfected with mito-Keima, incubated with knockdown or overexpression of IRF1 for 48 h, and then observed by fluorescence microscopy. Scale bars: 100 µm. (F) The relative ratio of red to green fluorescence area per cell of (E) was quantified. (G, H) ROS staining and quantification analysis in PHCs following either knockdown or overexpression of IRF1. Scale bars: 200 µm. Data are presented as the mean ± SD. Paired *t*-test (B, D, F, H) was used for statistical analysis. **P* < 0.05, ***P* < 0.01, and ****P* < 0.001.


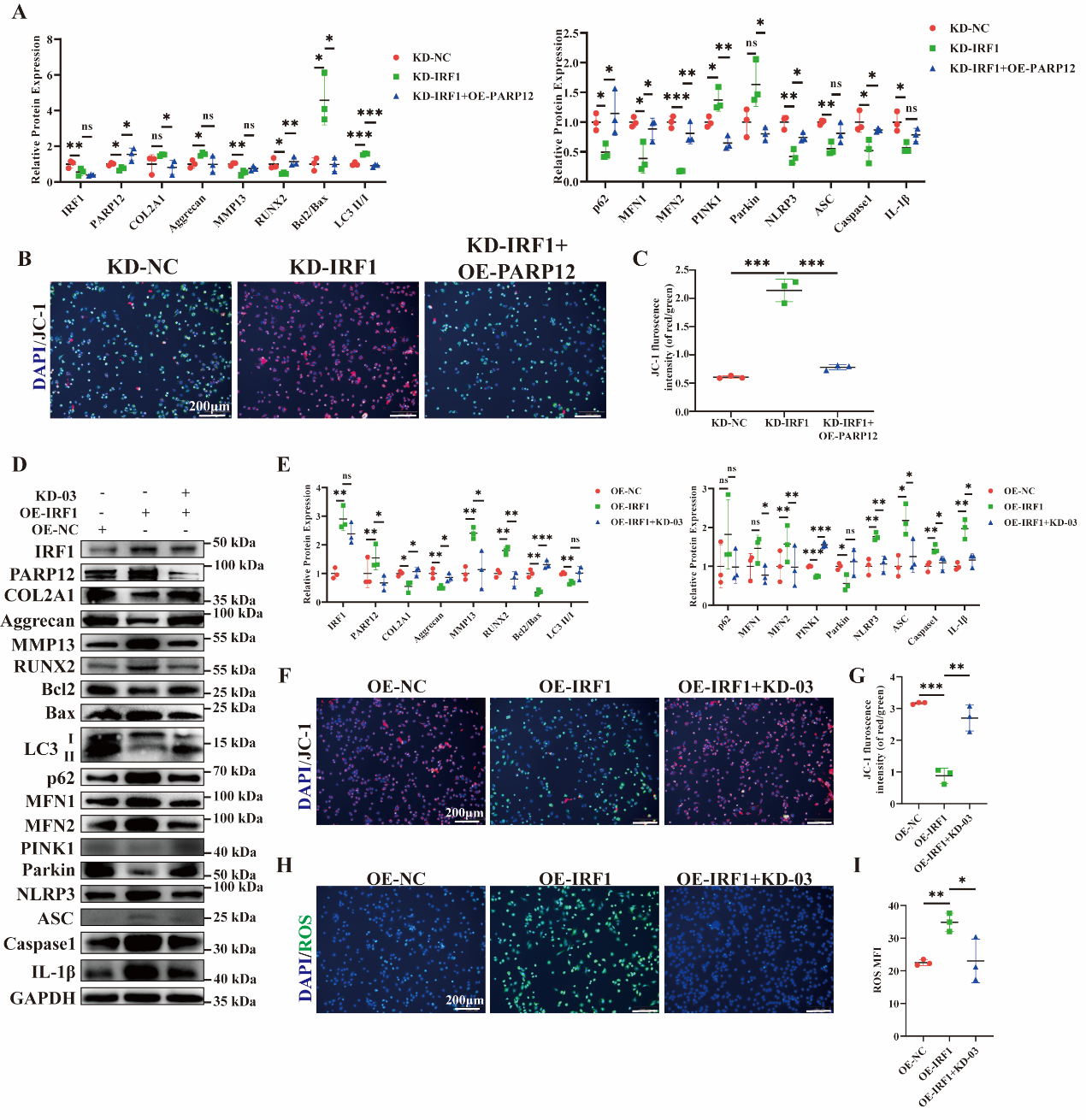


**Figure S9. The rescue experiment of IRF1 and PARP12.** (A) Protein quantification of IRF1, PARP12, COL2A1, Aggrecan, MMP13, RUNX2, Bcl2, Bax, LC3B II/I, p62, MFN1, MFN2, PINK1, Parkin and NLRP3 inflammasome activity with IRF1 knockdown and PARP12 overexpression. n=3 per group. (B) JC-1 staining in PHCs. Scale bars: 200 µm. (C) JC-1 staining quantification of (B) via ImageJ. n=3 per group. (D) Western blot analysis of IRF1, PARP12, COL2A1, Aggrecan, MMP13, RUNX2, Bcl2, Bax, LC3B II/I, p62, MFN1, MFN2, PINK1, Parkin and NLRP3 inflammasome activity with IRF1 overexpression and PARP12 knockdown. (E) Protein quantification of (D) via ImageJ. n=3 per group. (F) JC-1 staining in PHCs. Scale bars: 200 µm. (G) JC-1 staining quantification of (F) via ImageJ. n=3 per group. (H) ROS staining in PHCs. Scale bars: 200 µm. (I) ROS quantification of (H) via ImageJ. n=3 per group. Data were presented as mean ± SD. One-way analysis of variance followed by Tukey’s multiple comparison test (A, C, E, G, I) was used for statistical analysis. **P*<0.05, ***P*<0.01, ****P*<0.001.


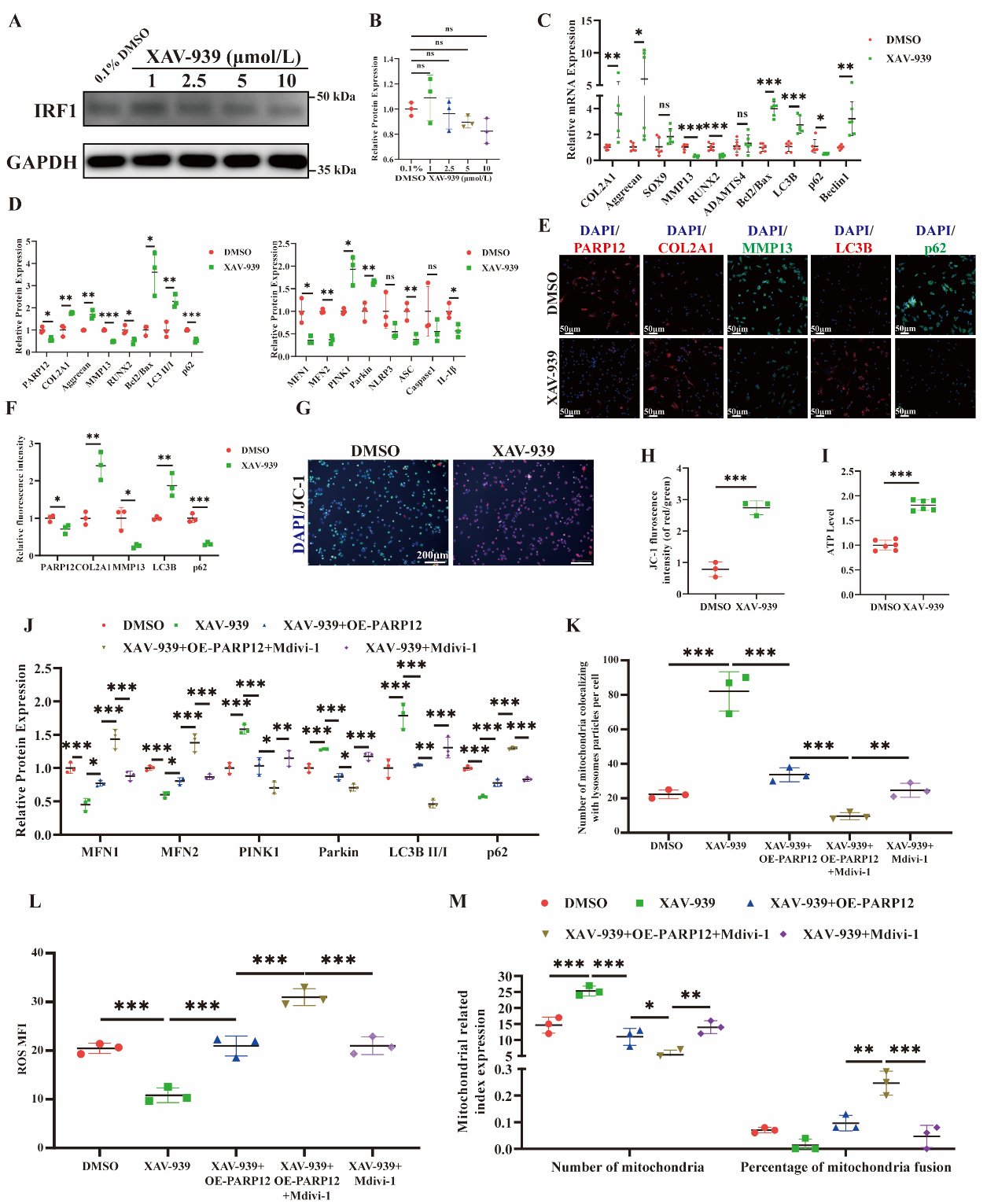


**Figure S10. XAV-939 promoted PINK1/Parkin-mediated mitophagy and suppressed cartilage degradation.** (A, B) Western blot and quantification analysis of IRF1 in PHCs treated with different concentrations of XAV-939. n=3 per group. (C) Quantitative PCR analysis of COL2A1, aggrecan, SOX9, MMP13, RUNX2, ADAMTS4, Bcl2/Bax, LC3B, p62, and Beclin1 in PHCs treated with XAV-939. n = 6 per group. (D) Protein quantification of PARP12, COL2A1, Aggrecan, MMP13, RUNX2, Bcl2/Bax, LC3B, p62, MFN1, MFN2, PINK1, Parkin and NLRP3 inflammasome activity via ImageJ. n=3 per group. (E) Immunofluorescence of PARP12, COL2A1, MMP13, LC3B, and p62 in PHCs treated with XAV-939. Scale bars: 50 µm. (F) Immunofluorescence quantification of (E) using ImageJ. n = 3 per group. (G) JC-1 staining in PHCs treated with XAV-939 or DMSO. Scale bars: 200 µm. (H) Quantification of JC-1 staining of (G) using ImageJ. n = 3 per group. (I) Quantification of the ATP level. n = 6 per group. (J-M) Quantification of Western blot analysis, TEM and ROS staining. n = 3 per group. Data were presented as mean ± SD. One-way analysis of variance with Dunnett’s multiple comparisons test (B, H-J) and paired *t*-test (C, D, F, H, I) were used for statistical analysis. **P* < 0.05, ***P* < 0.01, and ****P* < 0.001.


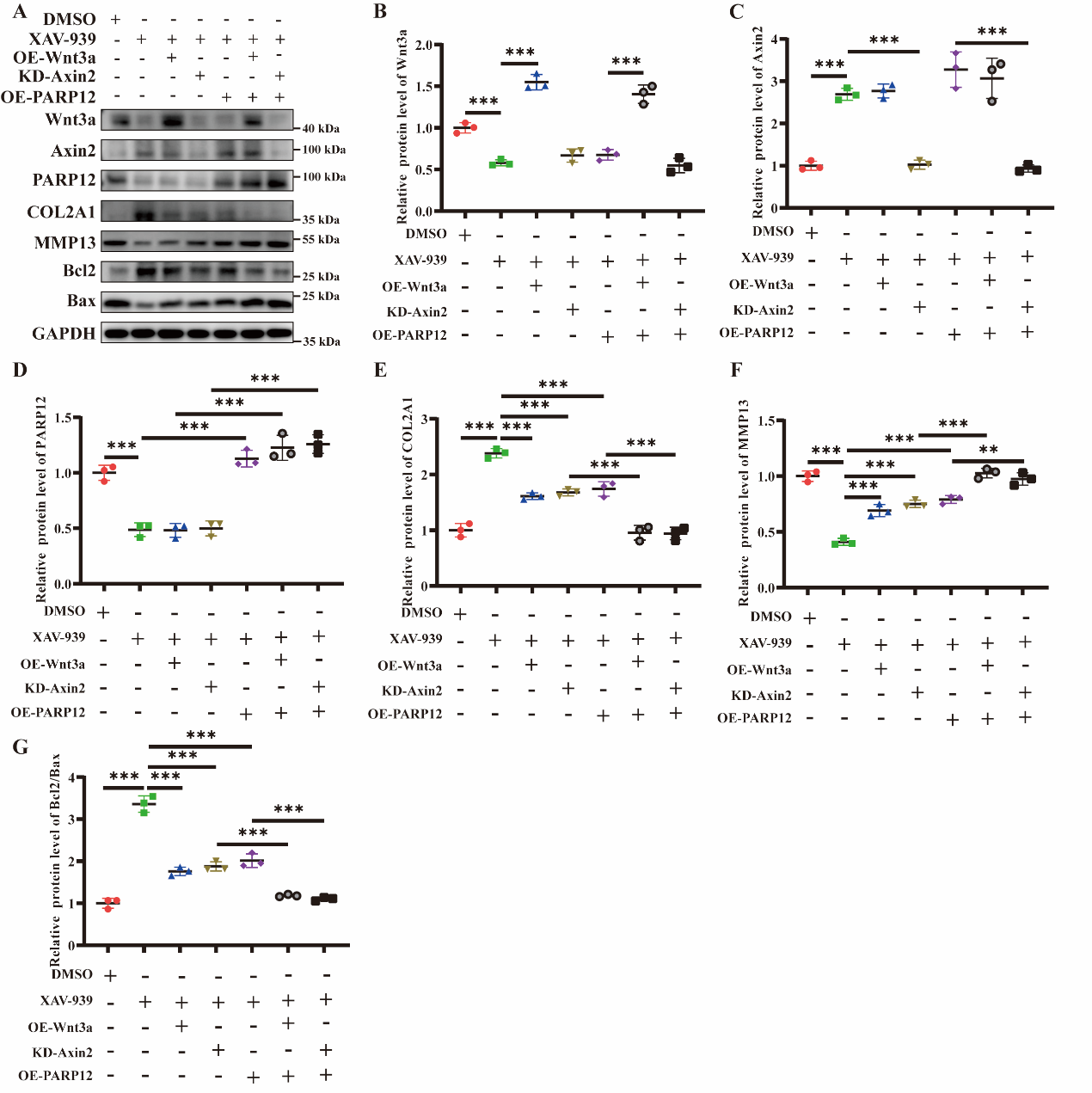


**Figure S11. XAV-939 could suppress cartilage degradation via targeting PARP12 besides inhibiting Wnt signaling pathway or promoting Axin2.** (A) Western blot analysis of Wnt3a, Axin2, PARP12, COL2A1, MMP13, Bcl2 and Bax in PHCs treated with XAV-939 or DMSO and transfected with Wnt3a-OE adenovirus, Axin2 siRNA or PARP12-OE adenovirus. (B-G) Protein quantification of (A) using ImageJ. n = 3 per group. One-way analysis of variance with Dunnett’s multiple comparisons test was used for statistical analysis. **P* < 0.05, ***P* < 0.01, and ****P* < 0.001.


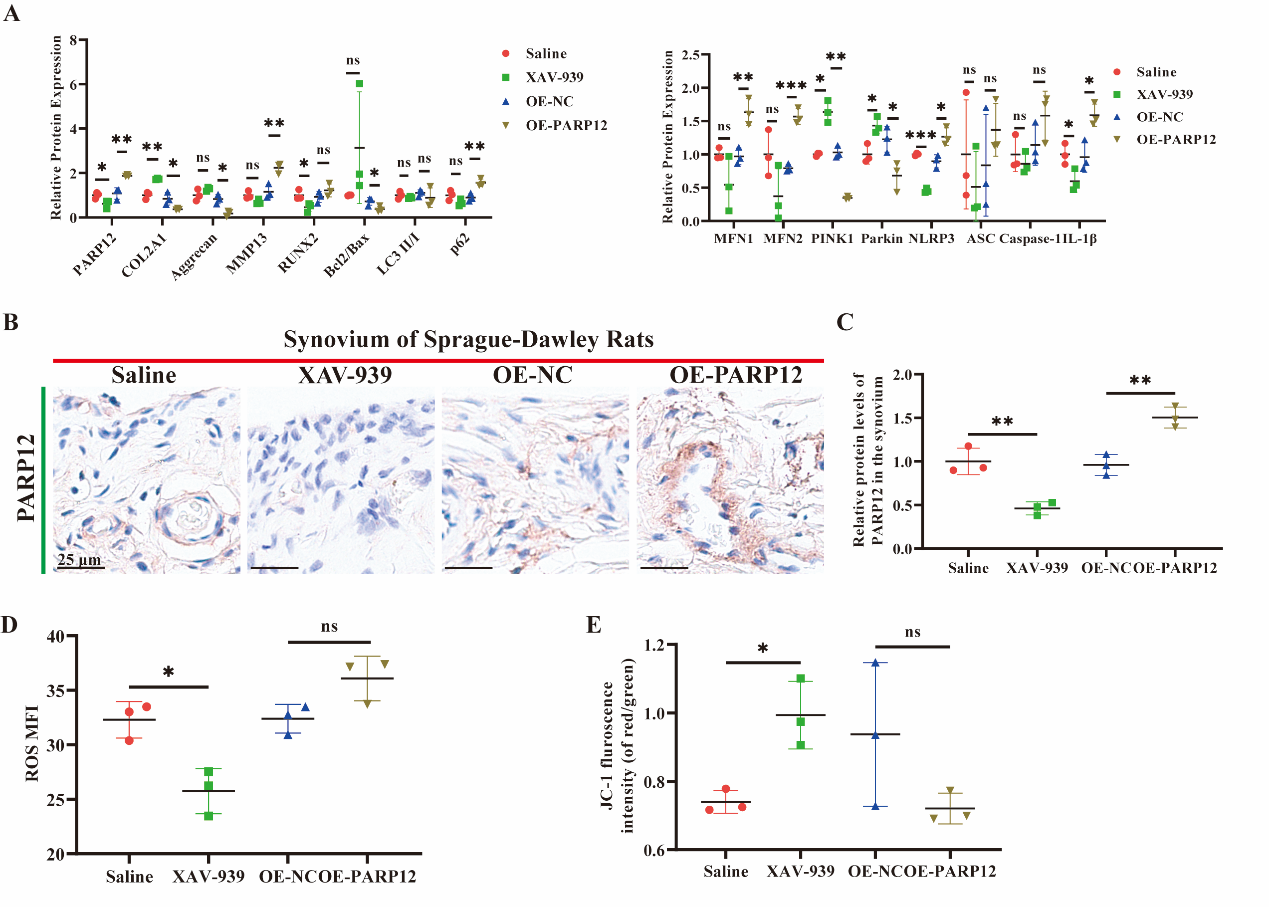


**Figure S12. PARP12 modulated osteoarthritis pathogenesis in rats.** (A) Protein quantification of PARP12, COL2A1, Aggrecan, MMP13, RUNX2, Bcl2/Bax, LC3B, p62, MFN1, MFN2, PINK1, Parkin and NLRP3 inflammasome activity via ImageJ. n=3 per group. (B, C) IHC staining and quantification analysis of PARP12 in synovium of Sprague–Dawley rats. Scale bars: 25 µm. n=3 per group. (D) Quantification of ROS staining using ImageJ. n=3 per group. (E) Quantification of JC-1 staining using ImageJ. n=3 per group. Data are presented as the mean ± SD. Paired *t*-test (A, C-E) was used for statistical analysis. **P* < 0.05, ***P* < 0.01, ****P* < 0.001.
